# Modulated Expression of the Protein Kinase GSK3 in Motor and Dopaminergic Neurons Increases Female Lifespan in *Drosophila melanogaster*

**DOI:** 10.3389/fgene.2020.00668

**Published:** 2020-06-30

**Authors:** Mikhail V. Trostnikov, Ekaterina R. Veselkina, Anna V. Krementsova, Stepan V. Boldyrev, Natalia V. Roshina, Elena G. Pasyukova

**Affiliations:** ^1^Laboratory of Genome Variation, Institute of Molecular Genetics, Russian Academy of Sciences, Moscow, Russia; ^2^Laboratory of Kinetics and Mechanisms of Enzymatic and Catalytic Reactions, N. M. Emanuel Institute of Biochemical Physics, Russian Academy of Sciences, Moscow, Russia; ^3^Laboratory of Genetic Basis of Biodiversity, N. I. Vavilov Institute of General Genetics, Russian Academy of Sciences, Moscow, Russia

**Keywords:** glycogen syntase kinase 3, transcription, lifespan, motor neurons, dopaminergic neurons, *Drosophila melanogaster*

## Abstract

Most eukaryotic genes express multiple transcripts and proteins, and a sophisticated gene expression strategy plays a crucial role in ensuring the cell-specificity of genetic information and the correctness of phenotypes. The *Drosophila melanogaster* gene *shaggy* encodes several isoforms of the conserved glycogen synthase kinase 3 (GSK3), which is vitally important for multiple biological processes. To characterize the phenotypic effects of differential *shaggy* expression, we explored how the multidirectional modulation of the expression of the main GSK3 isoform, Shaggy-PB, in different tissues and cells affects lifespan. To this end, we used lines with transgenic constructs that encode mutant variants of the protein. The effect of *shaggy* misexpression on lifespan depended on the direction of the presumed change in GSK3 activity and the type of tissue/cell. The modulation of GSK3 activity in motor and dopaminergic neurons improved female lifespan but caused seemingly negative changes in the structural (mitochondrial depletion; neuronal loss) and functional (perturbed locomotion) properties of the nervous system, indicating the importance of analyzing the relationship between lifespan and healthspan in invertebrate models. Our findings provide new insights into the molecular and cellular bases of lifespan extension, demonstrating that the fine-tuning of transcript-specific *shaggy* expression in individual groups of neurons is sufficient to provide a sex-specific increase in survival and slow aging.

## Introduction

According to both genome annotation data and experimental evidence, most *Drosophila melanogaster* genes express multiple transcripts and proteins^1^. Although little is known about why such sophisticated organization of gene structure and expression strategy is needed, it becomes clear that transcriptome burdening may play a crucial role in the implementation of information encoded in the genome. For example, there are many indications of significant sex-specific usage of alternative splicing and sex-specific transcription in *D*. *melanogaster* (Jin et al., 2001; Ranz et al., 2003; Telonis-Scott et al., 2009), and most sex-specific splicing is restricted to the gonads (Brown et al., 2014). Extensive alternative promoter usage and a great number of splicing events have been found in nervous tissue (Brown et al., 2014). It was demonstrated that most transcriptionally complex genes play significant tissue- and sex-specific roles (Huang et al., 2015). The exact and excessive regulation of gene expression reflects evolutionary and functional similarity between *D*. *melanogaster* and higher organisms such as mammals, which makes the fruit fly a practical model for functional genomics studies.

Elucidating the expression strategy and general gene biology is essential for understanding the genetic control of complex traits such as lifespan. The *D*. *melanogaster* gene *shaggy* (*sgg*) encodes the highly conserved serine-threonine protein kinase glycogen synthase kinase 3 (GSK3), a single homolog of human GSK3-beta, which governs multiple regulatory pathways and metabolic processes via interactions with InR/TOR, Wnt, JNK and other signaling cascades (McCubrey et al., 2016; Patel and Woodgett, 2017). Differential expression, that is, specific levels of suppression or enhancement of the expression of certain gene transcripts in certain tissues, allows a multiplicity of specific *sgg* functions. It was shown that the ubiquitous overexpression of *sgg* decreased lifespan and that ubiquitous *sgg* RNAi knockdown increased lifespan (Castillo-Quan et al., 2016). Later, we demonstrated that the misexpression of *sgg* affected lifespan in a transcript-, stage-, tissue- and sex-specific mode (Trostnikov et al., 2019). For example, the overexpression of the *sgg-RB* transcript (GenBank^2^ # AY122193.1; encodes the major GSK3 isoform (Ruel et al., 1993; Bourouis, 2002), in the nervous system caused pathological changes in neurons paralleled by a rapid decline in survival and severe shortening of lifespan, whereas *sgg-RB* overexpression in muscles caused only a weak decrease in survival. Overall, in Trostnikov et al. (2019), we presented data on the negative effects of strong GSK3 misexpression on lifespan, with the emphasis on the role of the pan-neuronal GSK3 misexpression as a cause of accelerated aging and neuronal pathology.

To further our research on the role of differential *sgg* expression in the control of lifespan, we suggested that the fine-tuning of tissue-specific *sgg* expression might provide an increase in survival and slow aging. Considering that finding the genetic bases of lifespan and healthspan extension continues to be an important and challenging goal of modern science, we decided to test this assumption. To change *sgg*/GSK3 expression, we used several independent methods, including overexpression of mutant *sgg* sequences, *sgg* knockdown, and knockdown of the gene encoding a GSK3 regulator, protein kinase aPKC, which allowed us to verify conclusions based on single experiments. In this paper, we presented our new data on positive effects of moderate levels of *sgg*/GSK3 misexpression on lifespan and demonstrated that these effects specifically depend on the cell type.

## Materials and Methods

### Fly Strains and Crosses

To provide mutant *sgg* overexpression, two lines were obtained from the Bloomington *Drosophila* Stock Center (USA)^3^ : *w[1118]; P{w* + *mC* = *UAS-sgg.Y214F}2* (in short, sgg-RB Y214F) with the transgenic construct encoding the PB form of GSK3 with an amino acid substitution Y214F in the activation loop, which is expected to reduce protein activity by about 20 times (Bourouis, 2002); and *w[1118]; P{w[*+ *mC]* = *UAS-sgg.A81T}MB2* (in short, sgg-RB A81T) with the transgenic construct encoding the PB form of GSK3 with an amino acid substitution A81T in the kinase domain, which is expected to block protein activity almost completely and causes dominant negative effect (Bourouis, 2002). *w[1118]* line without transgenic insertions was used as a control line (Bourouis, 2002).

To provide *sgg* RNAi knockdown, two lines were obtained from the Bloomington *Drosophila* Stock Center: *y^1^ sc^∗^ v^1^; P{y^+t7.7^ v^+t1.8^* = *TriP. HMS01751}attP40* (in short, sggKD1, with VALIUM20, hairpin size 21 bp, affects all *sgg* transcripts) and *y^1^ v^1^; P{y^+t7.7^ v^+t1.8^* = *TriP. JF01255}attP2* (in short, sggKD2, with VALIUM1, hairpin size 400 bp, affects all *sgg* transcripts). *y^1^ v^1^; P{*y^+t7^.^7^ = *CaryP}attP40* and *y^1^ v^1^; P{y^+t7.7^* = *CaryP}attP2* lines without transgenes providing RNAi were used as control lines for *sgg* RNAi knockdowns, as suggested by the manufacturer^4^.

To provide *aPKC* RNAi knockdown, two lines were obtained from the Bloomington *Drosophila* Stock Center: *y[1] sc[^∗^] v[1]; P{y[* + *t7.7] v[* + *t1.8]* = *TRiP.HMC06305}attP40* (in short, aPKC KD1, with VALIUM20, affects all transcripts) and *y[1] sc[^∗^] v[1]; P{y[* + *t7.7] v[* + *t1.8]* = *TRiP.HMS01689}attP40* (in short, aPKC KD2, with VALIUM20, affects all transcripts). *y[1] v[1]; P{y[* + *t7.7]* = *CaryP}attP40* line without transgenes providing RNAi was used as a control line, as suggested by the manufacturer^4^.

To induce overexpression or knockdown, several driver lines were obtained from the Bloomington *Drosophila* Stock Center.

*y[1] w^∗^; P{w* + *mW.hs* = *en2.4-GAL4}e22c; P{w* + *mC* = *tGPH}4/TM3, Ser[1]* (D1) was used to induce the expression of transgenic constructs in embryos.

*P{w* + *mC* = *UAS-Dcr-2.D}1, w[1118]; P{w* + *mC* = *GAL4-Mef2.R}R1* (D2) was used to induce the expression of transgenic constructs in somatic muscle cells.

*w^∗^; P{w* + *mC* = *ppl-GAL4.P}2* (D3) was used to induce the expression of transgenic constructs in the fat body.

*P{w* + *mW.hs* = *GawB}elavC155 w[1118]; P{w* + *mC* = *UAS-Dcr-2.D}2* (D4) was used to induce the expression of transgenic constructs in the nervous system.

*w^∗^; P{w* + *mW.hs* = *GawB}386Y* (D5) was used to induce the expression of transgenic constructs in peptidergic neurons.

*w^∗^; P{w* + *mC* = *ChAT-GAL4.7.4}19B* (D6) was used to induce the expression of transgenic constructs in cholinergic neurons.

*w[1118]; P{w* + *mW.hs* = *GawB}VGlutOK371* (D7) was used to induce the expression of transgenic constructs in glutamatergic neurons.

*P{w* + *mC* = *Gad1-GAL4.3.098}2/CyO* (D8) was used to induce the expression of transgenic constructs in GABAergic neurons.

*w^∗^; P{GawB}D42* (D9) was used to induce the expression of transgenic constructs in motoneurons.

*w[1118]; P{w* + *mC* = *Ddc-GAL4.L}Lmpt4.36* (D10) and *w^∗^; P{w[*+ *mC]* = *UAS-mCD8:GFP.L}LL5/Cy; P{w* + *mC* = *ple-GAL4.F}3* (D11) were used to induce the expression of transgenic constructs in dopaminergic neurons.

Additionally, the driver line *w[1118]; P{w* + *mC* = *UAS-mito-HA-GFP.AP}2/CyO; P{GawB}D42* (D12) obtained form S. V. Sarantseva (Sarantseva et al., 2012) was used to induce GFP expression in mitochondria of motor neurons.

D1, D2 and D4 driver lines proved to be effective according to the real time RT-qPCR data (Rybina et al., 2017, 2019).

To induce expression of transgenic constructs, females of each of the driver lines were crossed to males of sgg-RB Y214F, sgg-RB A81T, aPKC KD1, aPKC KD2, and Control lines. In all experiments, flies were kept at 25°C in glass vials measuring 25 by 100 mm on a medium containing 36 g of semolina, 40 g of sugar, 40 g of shredded raisins, 60 g of food yeast and 6 g agar (900g/cm2 gel strength) with 83 mg of streptomycin, 0.8 ml of propionic acid and nipagin (methylparaben) solution up to 0.04% per 1 liter. Approximately 10-15 males and females per vial were used for maintaining fly cultures and setting crosses.

### Tests for *Wolbachia*

As described in Trostnikov et al. (2019), prior to the experiments, all the lines were checked for the presence of *Wolbachia*, a *D*. *melanogaster* symbiont known to affect life history traits (McGraw and O’Neill, 2004), via quantitative PCR (MiniOpticon real-time PCR detection system, Bio-Rad) with primers for the 16S rRNA gene, 5′-CATACCTATTCGAAGGGATAG-3′ and 5′-AGCTTCGAGTGAAACCAATTC-3′ (Werren and Windsor, 2000). Negative results were obtained for all lines except D2 and D9. These lines were treated with tetracycline (0.25 mg/mL, Holden et al., 1993) for three generations followed by at least three generations of recovery, before they were used in experiments.

### Lifespan Assay

Lifespan was measured as described by Roshina et al. (2014) and Trostnikov et al. (2019). Five virgin flies of the same genotype and sex, all collected on the same day from cultures with moderate density, were placed in replicate vials. Flies were transferred to vials with fresh food containing approximately 5 ml of standard medium without live yeast on the surface weekly. The number of dead flies was recorded daily. Experiments comparing fly lifespans were conducted simultaneously. Sample sizes were 50–100 flies per sex per genotype. The experiments that showed noteworthy results were repeated two to three times with an interval of approximately 6 months. The lifespan for each fly was estimated as the number of days alive from the day of eclosion to the day of death. Mean lifespans and survival curves were primarily used to characterize lifespan.

### Locomotion Assay

Locomotion was measured as described by Roshina et al. (2014) and Trostnikov et al. (2019). Flies were collected and maintained by the same procedures as for the lifespan assays but without recording the deaths. Locomotion was measured, at the same time each day, in unmated males and females at age 1–3, 3–5, 20, 40, and 50 days. Experiments comparing locomotion were conducted simultaneously. Sample sizes were 30–155 flies (6–31 vials) per sex per genotype; reduced sample sizes were typical for experiments with old flies. To measure locomotor activity, the vials were placed horizontally in a *Drosophila* Population Monitor (TriKinetics). Fly movement along the walls or in the middle of the vial crossed the infrared beam rings along the length of the vial. Beam interruptions were detected and totals were reported every 5 min to the host computer. Two measurements for 5 min were made for each vial. Locomotion was characterized as the mean number of beam interruptions per vial.

### Real-Time RT-qPCR

As described in Trostnikov et al. (2019), total RNA for real-time reverse transcription quantitative PCR (RT-qPCR) was extracted from batches of 30 whole bodies of 3–days old males and females using TRIzol reagent (Invitrogen) and DNase I (Sigma-Aldrich) according to the manufacturers’ instructions.

First-strand cDNA was synthesized using SuperScript II Reverse Transcriptase (Invitrogen) with oligo(dT)15 primers according to the manufacturer’s instructions. Amounts of cDNA were determined by RT-qPCR using SYBR Green I in a MiniOpticon real-time PCR detection system (Bio-Rad).

Primers were specifically designed to detect only *sgg-RB* and not any of the other *sgg* transcripts: shaggyPB1 5′-ATA TACAGATCTTTTGTTTGGCAA-3′ and shaggyPB2 5′-AGGA GGAAGTTCTTGGACGA-3′. *Gdh* and *Adh* housekeeping genes, characterized by moderate expression comparable to *sgg* expression, were used as reference genes to normalize for differences in total cDNA between the samples. The forward and reverse primers for the reference genes were as follows: for *Gdh*, Gdh1 5′-TATGCCACCGAGCACCAGATTCC-3′ and Gdh2 5′-GGATGCCCTTCACCTTCTGCTTCTT-3′; for *Adh*, Adhd3: 5′-CGGCATCTAAGAAGTGATACTCCCAAAA-3′ and Adhr3: 5′-TGAGTGTGCATCGAATCAGCCTTATT-3′.

CFX Manager 3.1 software (Bio-Rad, 2012) was used to evaluate the relative gene expression. Inter-run calibrations were used for each panel of experiments since the experiments were conducted for several years. Two independent RNA extractions (biological replicates) per sex per genotype and three technical repeats for each RNA extraction were made.

### Western Blotting

As described in Trostnikov et al. (2019), for evaluation of the protein amount in motor neurons and the brains, approximately 30 thoraxes and 30 heads, respectively, of 3–5 days old adults of each genotype were dissected and homogenized in 8 M urea solution. Equal amounts of samples from the supernatants were preincubated with sample buffer (deionized water, 0.5 M Tris-HCl, glycerol, 10% SDS, 0.5% bromphenol blue, DTT) for 5 min at 95°C and separated in a 4–12% (w/v) acrylamide Bis/Tris SDS-PAGE gel using the vertical electrophoretic chamber Mini-Protean Tetra (Bio-Rad). Proteins were transferred from the gel to the PVDF membrane (Immobilon-P Membrane) using electroblotting (Mini Trans-Blot Modul, Bio-Rad), blocked in BlockPro blocking buffer (Visual Protein) and incubated with anti–GSK3 beta primary antibodies (1:300; ab18893, Abcam) for 1 h. Bound antibodies were detected with goat anti–rabbit secondary antibodies conjugated with alkaline phosphatase (1:20000; A3687, Sigma). Prior to visualization, the membranes were incubated in the alkaline CDP buffer for 5 min and then in the Immun-Star AP- Substrate (Bio-Rad) for 7 min. After scanning, the relative intensity quantification of each band was evaluated with Image Lab software (Bio-Rad). Three to four independent protein extractions (biological replicates) per sex per genotype were made.

### Immunostaining and Microscopy

As described in Trostnikov et al. (2019), body wall muscles and neurons of male and female third-stage larvae and brains of 3–5 days old unmated females were dissected in phosphate-buffered saline (PBS), fixed in 4% paraformaldehyde (Sigma-Aldrich) at room temperature for 20 min, and washed in PBS (3 × 15 min). For immunostaining, preparations were blocked in blocking buffer (BlockPRO, Visual Protein Biotechnology Corporation, US) for 1 h at room temperature, incubated in primary antibodies (diluted in BlockPRO) overnight at 4°Ñ, washed in PBS (3 × 15 min), incubated in secondary antibodies (diluted in BlockPRO) for 2 h, washed in PBS (3 × 15 min) and placed in a medium for immunofluorescence (VectaShield, Vector Labs). Neuromuscular junctions (NMJs) were analyzed in the fourth muscle of the third and fourth abdominal segments of larvae. A confocal laser scanning microscope (LSM 510, Zeiss), ImageJ^5^ and LSM Image Browser (Zeiss) were used. Sample sizes were 8–16 specimens per genotype per experiment. The mean number of synaptic active zones was used to characterize synapse activity. Mean numbers of mitochondria and dopaminergic neurons were calculated.

The following primary antibodies were used: mouse anti-Brp [mAb NC82, 1:200; Developmental Studies Hybridoma Bank (DSHB)] against Bruchpilot (BRP), a protein specific to active synaptic zones (Wagh et al., 2006); Alexa Fluor 647-conjugated goat anti-HRP (1:400, Jackson ImmunoResearch), against Horseradish Peroxidase (HRP), a widely used marker of presynaptic membranes (Franco et al., 2004). The secondary antibodies used were goat anti-mouse Cy3 conjugated (1:400, Jackson ImmunoResearch). Antibodies obtained from the DSHB were developed under the auspices of the NICHD and maintained by The University of Iowa, Department of Biology, Iowa City, IA 52242. Dopaminergic neuron were visualized with GFP using the D11 driver line. Mitochondria were visualized with GFP using the D12 driver line.

### Statistical Analyses

To compare control and mutant genotypes, Student’s *t*-test and the non-parametric, distribution-free Kruskal-Wallis test were used for analyses of GSK3 amounts; locomotion; active zones and mitochondria in NMJs. These two tests gave consistent results, so only the results of the Kruskal-Wallis test are reported here. Standard descriptive statistical analysis of lifespan (Wilmoth and Horiuchi, 1999; Carey, 2003) was performed to determine the mean lifespan and its accompanying variances, standard deviations and standard errors; the median, minimum and maximum lifespans; and the lifespans of the lower and upper quartiles, 10 and 90 percentiles (Supplementary Tables S1, S3, S4). Survival curves were estimated using the Kaplan–Meier procedure. The non-parametric, distribution-free Mann-Whitney test and Kolmogorov-Smirnov test were used to evaluate the statistical significance of the difference between the survival curves. The Wang–Allison test was used to evaluate the statistical significance of differences at the age of 25 and 90% mortality (Wang et al., 2004). The initial (R_0_) and age-dependent (α) mortality (parameters of the Gompertz equation (μ(x) = R_0_e^α*x*^, where μ(x) is survival at age x)) and the mortality rate doubling time (MRDT = ln2/α) were estimated. Statistical analysis was carried out using *Statistica* version 13.5, Tibco Software Inc. and OASIS 2: Online Application for Survival Analysis 2 (Han et al., 2016).

## Results

### The Model System

To ensure a moderate change in *sgg* expression, we decided to use existing genetic tools. In 2002, Bourouis described several transgenic constructs containing normal or mutant *sgg-RB* cDNA.

One of these mutants (*sgg-RB Y214F*) encodes a protein with a Y214F amino acid substitution in the activation loop, which is assumed to reduce GSK3 activity by 20 times (Bourouis, 2002). This assumption is based on information on the properties of the orthologous Y216F substitution in the sequence of the human protein. Data on the magnitude of the effect of the Y216F substitution on protein activity are somewhat controversial (Lochhead et al., 2006, and references within), but it is evident that phosphorylated Y216/214 is essential for full enzyme activity in humans (Lochhead et al., 2006) and for any physiological function in *D*. *melanogaster* (Papadopoulou et al., 2004). The Y216F substitution may promote the formation of low affinity homodimers of GSK3, which can only form from the protein unphosphorylated at the Y216 site and are predicted to be catalytically inactive (Harwood, 2001; Fraser et al., 2002). The formation of inactive dimers may explain a decrease in the activity of the total GSK3 pool; it remains unclear, however, whether GSK3 forms dimers *in vivo*. Due to the suggested reduced kinase activity of GSK3 with the Y214F substitution, the overexpression of *sgg-RB Y214F* mutant copy of *sgg* is expected to increase total GSK3 activity to a lesser extent than the overexpression of normal *sgg*, that is, to moderately increase total GSK3 activity. Although the evidences for changes in GSK3 activity in *D*. *melanogaster* are indirect, hereinafter, the effect of overexpression of *sgg-RB Y214F* will be referred to as moderate overexpression of *sgg* or a moderate increase in GSK3 activity.

The other mutant (*sgg-RB A81T*) has an A81T amino acid substitution in the kinase domain, which is supposed to block GSK3 activity (Bourouis, 2002). In humans, residues which form the ATP binding site (for example, K85) are commonly mutated to generate kinase-dead variants. The failure of binding target proteins arises probably due to the disruption of the correct folding of GSK3 (Fraser et al., 2002). The overexpression of *sgg-RB A81T* was shown to produce a dominant-negative effect (Bourouis, 2002; Franco et al., 2004; Franciscovich et al., 2008; Cuesto et al., 2015), that is, to moderately decrease total GSK3 activity. It was suggested that dominant negative effects of kinase-deficient mutations of the conserved residues in the ATP binding region may be due to competition between mutant GSK3 and endogenous GSK3 for downstream targets or upstream regulators (Kaidanovich-Beilin and Woodgett, 2011). Although the evidences for changes in GSK3 activity are indirect, hereinafter, the effect of overexpression of *sgg-RB A81T* will be referred to as the dominant negative effect or a moderate decrease in GSK3 activity.

For comparison, we will use the data on the effect of overexpression of the normal copy of *sgg-RB* on lifespan and the nervous system (Trostnikov et al., 2019). The effect of overexpression of the normal copy of *sgg-RB* will be referred to as strong overexpression of *sgg* or a strong increase in GSK3 activity.

It was tempting to directly characterize total GSK3 activity in tissues upon *sgg-RB Y214F* and *sgg-RB A81T* overexpression. Such a measurement, however, is not technically feasible. First, the most commonly used method of detecting GSK3-beta activity in human tissues, that is, examining Ser9 phosphorylation, which activates the enzyme, is not suitable because changes in GSK3 activity are not always accompanied by changes in its phosphorylation status (Hur and Zhou, 2010). Second, the obvious approach of evaluating the phosphorylation status of GSK3 targets as a measure of GSK3 activity is hampered by the fact that GSK3 has many substrates, and it is not evident which of them is appropriate to study in different tissues. Finally, the *in vitro* kinase assay that is aimed to detect GSK3 activity on its substrates presumes that endogenous GSK3 can be immunoprecipitated from cell lysates. However, given that, in cells, GSK3 functionality is believed to be dependent of its subcellular compartmentalization (Patel and Woodgett, 2017), the analysis of total cell lysates may distort the results. In *D*. *melanogaster*, additional technical difficulties exist because of the poor availability of commercial antibodies specific for phosphorylated residues of both GSK3 and potential GSK3 substrates, as well as of specific substrates for the *in vitro* kinase assay.

Two lines expressing a transgenic construct that encodes the major PB isoform of GSK3 with an amino acid substitution, namely, sgg-RB Y214F (*w[1118]; P{w* + *mC* = *UAS-sgg. Y214F}2*) or sgg-RB A81T (*w[1118]; P{w[*+ *mC]* = *UAS-sgg. A81T}MB2*), and a control (*w[1118]*) line without transgenic insertions described in Bourouis (2002) were obtained from the Bloomington *Drosophila* Stock Center (USA)^3^. To characterize the effects of mild *sgg* misexpression on lifespan, we induced *sgg-RB Y214F* and *sgg-RB A81T* overexpression in embryos (the D1 driver line), in three tissues, namely, muscle (the D2 driver line), the fat body (the D3 driver line), and the nervous system (the D4 driver line), and in different neurons (D5–D11 driver lines) using the GAL4-UAS binary system (Brand and Perrimon, 1993). As the direction of crosses could be important for the results, in our study, we always used females from the driver lines. It follows that, in a given tissue, we always compared lifespans and other phenotypes in individuals with the same cytoplasm, but with different *sgg* genotypes. Thus, the maternal effect was fully taken into account.

As a result of screening, we were most interested in identifying cases of a positive effect of the *sgg* misexpression on lifespan. Given this, we repeated those experiments in which such an effect was detected in order to confirm it. The negative effect of *sgg* misexpression on lifespan was confirmed only for those experiments that we considered the most important; in other cases, a comparison of the results of moderate overexpression and the dominant negative effect with the results of strong overexpression and knockdown of *sgg* (Trostnikov et al., 2019) allowed us to obtain reliable conclusions.

### Screening for the Effects of Moderate Changes in *sgg* Expression on Lifespan

As stated previously (Trostnikov et al., 2019), our primary goal was to understand how *sgg* misexpression affects the nervous system, lifespan and aging. However, to obtain a broader view, we previously tested the effects of strong *sgg* overexpression and *sgg* knockdown in two other tissues and at the embryonic stage (Trostnikov et al., 2019). The fat body attracted our attention because it was repeatedly shown to play an important role in lifespan control (Giannakou et al., 2004; Bai et al., 2012; Hoffmann et al., 2013). In contrast, little is known about the role of muscles in controlling longevity (Rybina et al., 2017), and we considered misexpression in this tissue as a negative control for misexpression in the nervous system and in the fat body. Our particular interest in the role of embryonic *sgg* expression in controlling lifespan was related to the fact that, earlier, we demonstrated that changes in gene expression at the embryonic stage are responsible for increases in adult longevity (Roshina et al., 2014). To continue our research, we decided to determine how mild *sgg* misexpression in the nervous system, in the fat body, in muscles and at the embryonic stage affects lifespan.

**FIGURE 1:**
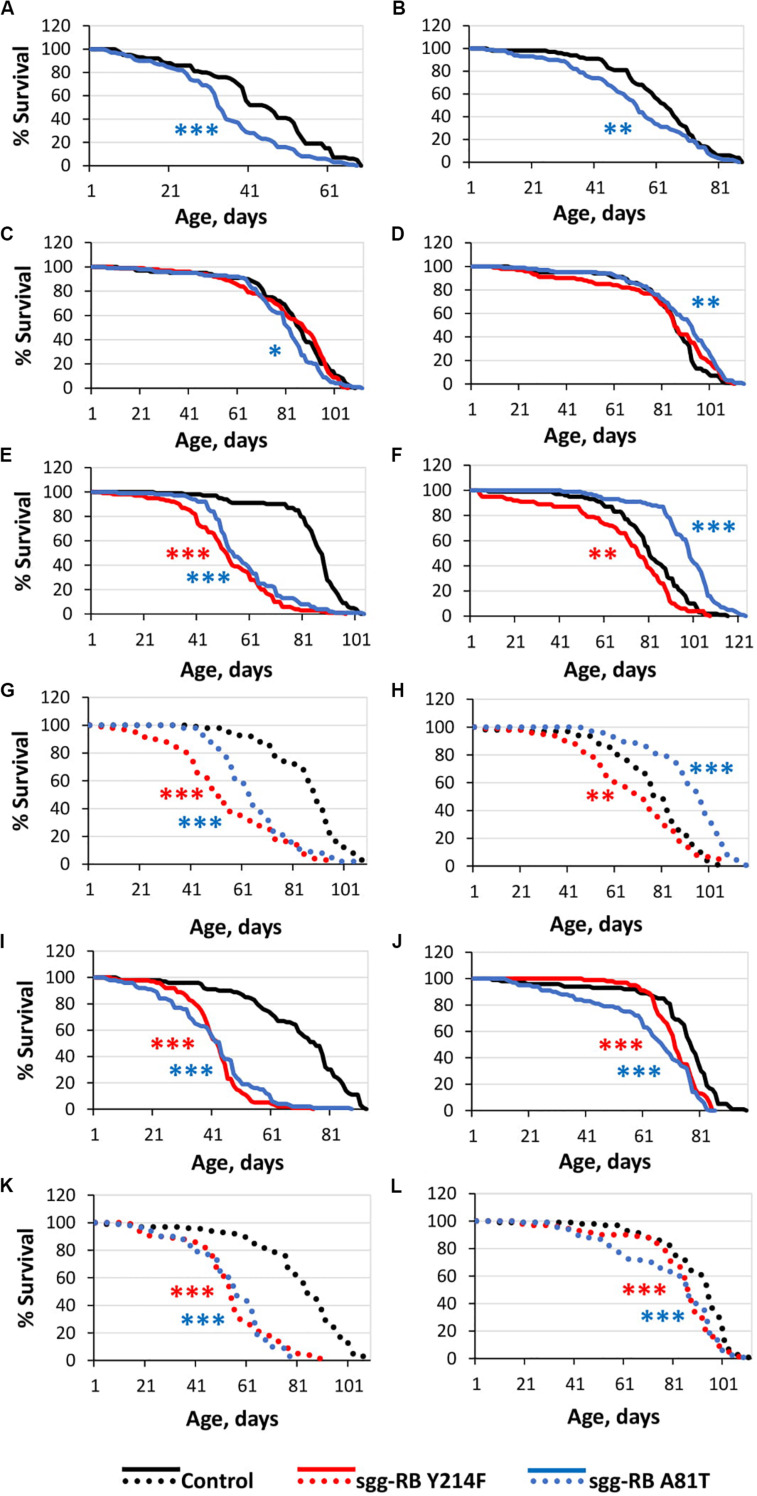
Effects of moderate changes in *sgg* expression in embryos **(A,B)**, muscle **(C,D)**, the fat body [**(E,F)** – first experiment, solid lines; **(G,H)** – second experiment, dotted lines] and the nervous system [**(I,J)** – first experiment, solid lines; **(K,L)** – second experiment, dotted lines] on male **(A,C,E,G,I,K)** and female **(B,D,F,H,J,L)** survival. Control, sgg-RB Y214F, and sgg-RB A81T denote hybrid genotypes obtained as a result of crossing *w[1118]*, *w[1118]; P{w* + *mC* = *UAS-sgg.Y214F}2* and *w[1118]; P{w[*+ *mC]* = *UAS-sgg.A81T}MB2* females, respectively, with *y[1] w^∗^; P{w* + *mW.hs* = *en2.4-GAL4}e22c; P{w* + *mC* = *tGPH}4/TM3, Ser[1]*, *P{w* + *mC* = *UAS-Dcr-2.D}1, w[1118]; P{w* + *mC* = *GAL4-Mef2.R}R1*, *w^∗^; P{w* + *mC* = *ppl-GAL4.P}2* and *P{w* + *mW.hs* = *GawB}elavC155 w[1118]; P{w* + *mC* = *UAS-Dcr-2.D}2* males to induce the expression of transgenic constructs in embryos, muscle, the fat body and the nervous system, respectively. *P < 0.05, **P < 0.01, and ***P < 0.001, as determined by the Mann-Whitney test.

**FIGURE 2:**
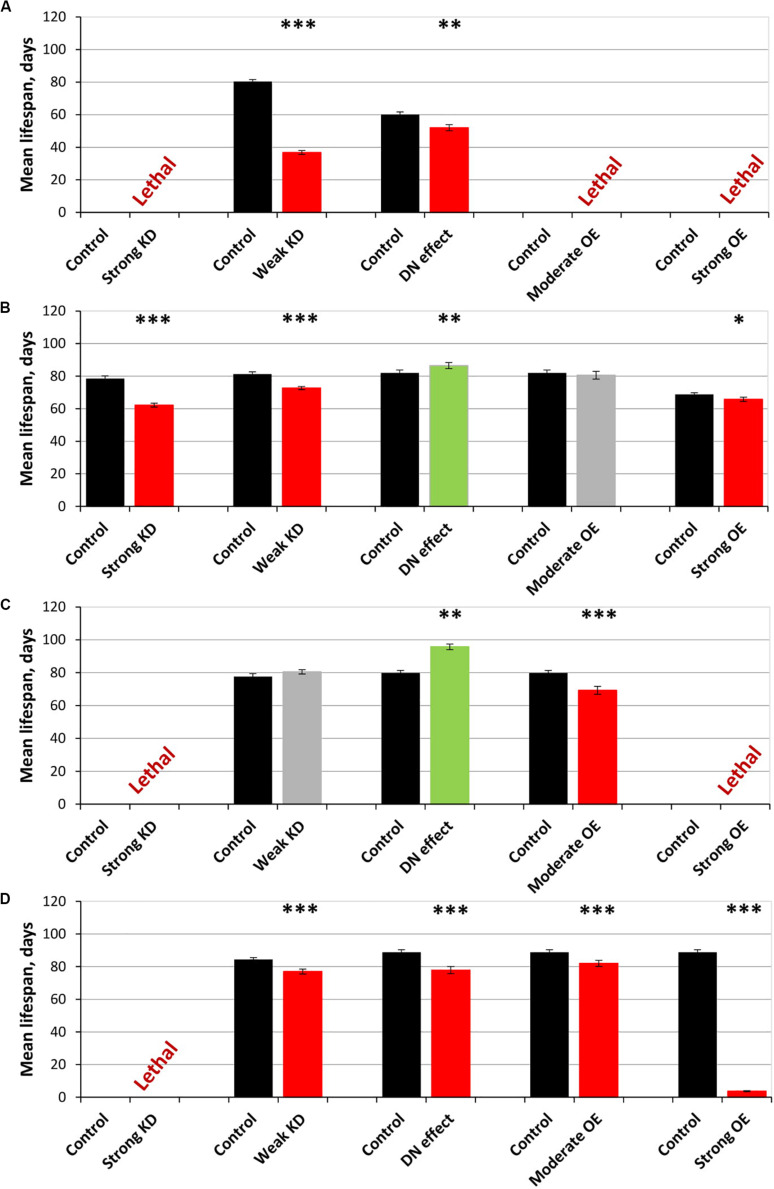
Effects of multidirectional changes in *sgg* expression in embryos **(A)**, muscle **(B)**, the fat body **(C)** and the nervous systemc **(D)** on the mean lifespan of females. Control and Strong knockdown (KD); Control and Weak KD; Control and Dominant negative (DN) effect; Control and Moderate overexpression (OE); Control and Strong OE denote hybrid genotypes obtained as a result of crossing 1) *y^1^ v^1^; P{*y^+t7^.^7^ = *CaryP}attP40* and *y^1^ sc^∗^ v^1^; P{y^+t7^.^7^ v^+t1^.^8^* = *TriP. HMS01751}attP40*; 2) *y^1^ v^1^; P{y^+t7^.^7^* = *CaryP}attP2* and *y^1^ v^1^; P{y^+t7^.^7^ v^+t1^.^8^* = *TriP. JF01255}attP2*; 3) *w[1118]* and *w[1118]; P{w[*+ *mC]* = *UAS-sgg.A81T}MB2*; 4) *w[1118]* and *w[1118]; P{w* + *mC* = *UAS-sgg.Y214F}2*; 5) *w[1118]* and *w[1118]; P{w* + *mC* = *UAS-sgg.B}*MB5 females, respectively, with *y[1] w^∗^; P{w* + *mW.hs* = *en2.4-GAL4}e22c; P{w* + *mC* = *tGPH}4/TM3, Ser[1]*, *P{w* + *mC* = *UAS-Dcr-2.D}1, w[1118]; P{w* + *mC* = *GAL4-Mef2.R}R1*, *w^∗^; P{w* + *mC* = *ppl-GAL4.P}2* and *P{w* + *mW.hs* = *GawB}elavC155 w[1118]; P{w* + *mC* = *UAS-Dcr-2.D}2* males to induce the expression of transgenic constructs in embryos, muscle, the fat body and the nervous system, respectively. ^∗^*P* < 0.05, ^∗∗^*P* < 0.01, and ^∗∗∗^*P* < 0.001, as determined by the Kruskal-Wallis test.

The overexpression of *sgg-RB Y214F* in embryos was lethal; the overexpression of *sgg-RB A81T* in embryos moderately reduced the lifespans of both males and females (Supplementary Table S1; Figures 1A,B). These effects were similar to the effects of the overexpression of normal *sgg-RB* and weak *sgg* knockdown, respectively, observed previously, but differed in the strength of the effect (Trostnikov et al., 2019; Figure 2A and Supplementary Figure S1A). These results were in good agreement with the suggestion that *sgg-RB Y214F* overexpression increases GSK3 activity and *sgg-RB A81T* overexpression decreases GSK3 activity.

The overexpression of the mutant *sgg-RB Y214F* in muscle had no effect on the lifespan of either males or females, and the overexpression of *sgg-RB A81T* very slightly though significantly decreased male lifespan and increased female lifespan (5% for both, Supplementary Table S1, Figures 1C,D, 2B, and Supplementary Figure S1B). The lack of notable effects indicated that lifespan was not particularly sensitive to moderate changes in GSK3 activity in muscle. The overexpression of normal *sgg-RB* and both strong and weak *sgg* knockdown in muscle decreased male and female lifespans to varying degrees (Trostnikov et al., 2019; Figure 2B, Supplementary Figure S1B). Interestingly, changes induced by knockdowns and various overexpressions lead to the appearance of a regular pattern of effects (Figure 2B and Supplementary Figure S1B), even though knockdowns affected all *sgg* transcripts, whereas *sgg* overexpressions affected only *sgg-RB*. These effects changed in accordance with the proposed alterations in GSK3 activity which varied from a strong decrease as a result of a strong *sgg* knockdown, to a lower decrease as a result of a weak *sgg* knockdown, to a still smaller decrease as a result of *sgg-RB A81T* overexpression, and then to a moderate and strong increase as a result of *sgg-RB Y214F* and *sgg-RB* overexpression, respectively.

The overexpression of *sgg-RB Y214F* in the fat body substantially decreased male lifespan, whereas in females, the negative effect was smaller (Supplementary Table S1, Figures 1E,F, 2C, and Supplementary Figure S1C). The overexpression of normal *sgg-RB* in the fat body was lethal both in males and in females (Trostnikov et al., 2019; Figure 2C and Supplementary Figure S1C). Such a result again indicated that the addition of mutant *sgg* presumably increased the activity of GSK3 to a lesser extent than the addition of normal *sgg*. The overexpression of *sgg-RB A81T* in the fat body substantially decreased male lifespan, whereas in females, it significantly increased lifespan (Supplementary Table S1, Figures 1E,F, 2C, and Supplementary Figure S1C). In the second experiment, which was aimed to verify the results, all the effects, including the positive effect in females, were reproduced (Supplementary Table S1 and Figures 1G,H). The weak *sgg* knockdown decreased male lifespan and did not statistically affect female lifespan; however, for female lifespan, the survival curve indicated a possible positive effect (Trostnikov et al., 2019; Figure 2C and Supplementary Figure S1C). Our observations were in good agreement with the suggestion that *sgg-RB A81T* overexpression decreases GSK3 activity. A plausible suggestion is, again, that the dominant-negative effect of *sgg-RB A81T* overexpression decreased GSK3 activity to a lesser extent than weak *sgg* knockdown and that this negative effect was favorable for female longevity. Accordingly, whereas knockdown led to a barely noticeable increase in female lifespan, the dominant-negative effect caused a substantial increase in female survival. Mean lifespan (20% average increase) and maximum lifespan (age of 90% mortality, 11% average increase) were significantly elevated (Supplementary Tables S1, S2). Survival curves were fitted by Gompertz function; comparison of the Gompertz function parameters showed that in females with *sgg-RB A81T* overexpression in the fat body, the initial level of mortality was lower in both experiments, whereas the age-dependent mortality rate was similar to the control mortality rate (Supplementary Table S2). Thus, we concluded that *sgg-RB A81T* overexpression in the fat body had a positive impact on both early mortality and maximum lifespan.

We have already shown that, in the nervous system, of all tested transcripts, only *sgg-RB* appeared to be functional (Trostnikov et al., 2019). The overexpression of *sgg-RB Y214F* and *sgg-RB A81T* in the nervous system reduced male lifespan to a similar extent, i.e., by approximately 35–40% (Supplementary Table S1, Figure 1I, and Supplementary Figure S1D); the effect in females was less pronounced (approximately 5 and 15%, respectively) (Supplementary Table S1 and Figures 1J, 2D). Given our special interest in the effects of GSK3 in the nervous system, we repeated our measurements and obtained very similar results (Supplementary Table S1 and Figures 1K,L). The strong *sgg* knockdown and overexpression of normal *sgg-RB* and in the nervous system were lethal or almost lethal, respectively (Trostnikov et al., 2019), whereas weak *sgg* knockdown (Trostnikov et al., 2019), overexpression of *sgg-RB A81T* and *sgg-RB Y214F* had similar and much smaller negative effects on lifespan (Figure 2D and Supplementary Figure S1D). Thus, the nervous system showed very strong sensitivity to severe changes in *sgg* expression and restrained sensitivity to multidirectional moderate changes in *sgg* expression.

The *elav* driver (D4) used in these experiments provides panneuronal expression^6^. The nervous system is composed of different types of neurons, and our previous results demonstrated that *sgg* misexpression in neurons with different functionality had varying in strength negative effects on lifespan (Trostnikov et al., 2019). To discriminate the effects of *sgg* misexpression in neurons that secrete different transmitters and in motor neurons on lifespan, we overexpressed *sgg-RB Y214F* and *sgg-RB A81T* using the neuron-specific drivers D5-D10 (see “Materials and Methods” section). No changes in male or female lifespan were observed when *sgg-RB Y214F* and *sgg-RB A81T* were overexpressed in peptidergic and cholinergic neurons (Supplementary Table S3 and Supplementary Figures S2A–D). The overexpression of both transgenes in glutamatergic and GABAergic neurons caused a significant decrease in male lifespan (Supplementary Table S3 and Supplementary Figures S2E,G) but did not affect female lifespan (Supplementary Table S3 and Supplementary Figures S2F,H).

**FIGURE 3:**
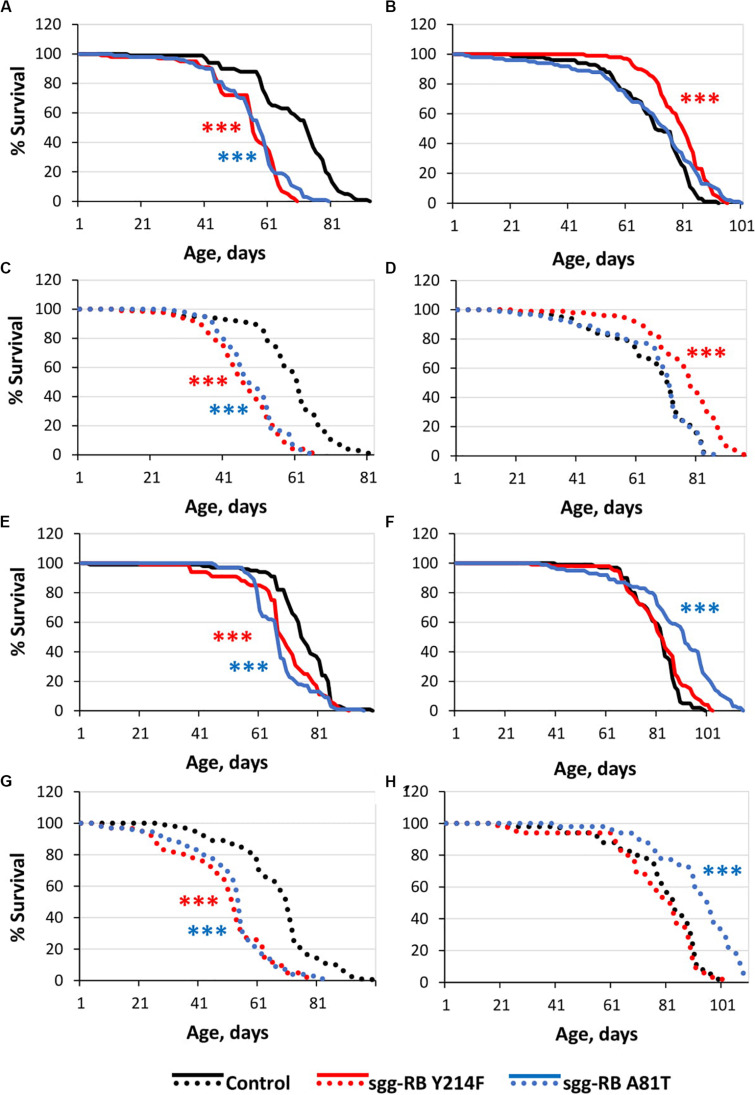
Effects of moderate changes in *sgg* expression in motor [**(A,B)** – first experiment, solid lines; **(C,D)** – second experiment, dotted lines] and dopaminergic [**(E,F)** – first experiment, solid lines; **(G,H)** – second experiment, dotted lines] neurons on male **(A,C,E,G)** and female **(B,D), (F,H)** survival. Control, sgg-RB Y214F, and sgg-RB A81T denote hybrid genotypes obtained as a result of crossing *w[1118]*, *w[1118]; P{w* + *mC* = *UAS-sgg.Y214F}2* and *w[1118]; P{w[*+ *mC]* = *UAS-sgg.A81T}MB2* females, respectively, with *w^∗^; P{GawB}D42* and *w[1118]; P{w* + *mC* = *Ddc-GAL4.L}Lmpt4.36* males to induce the expression of transgenic constructs in motor and dopaminergic neurons, respectively. ***P < 0.001, as determined by the Mann-Whitney test.

**FIGURE 4:**
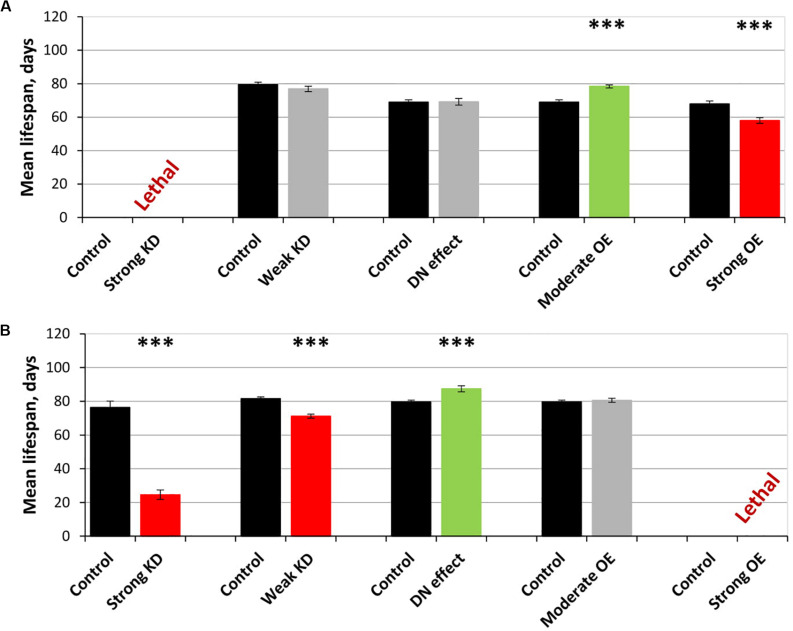
Effects of multidirectional changes in *sgg* expression in motor **(A)** and dopaminergic **(B)** neurons on the mean lifespan of females. Control and Strong knockdown (KD); Control and Weak KD; Control and Dominant negative (DN) effect; Control and Moderate overexpression (OE); Control and Strong OE denote hybrid genotypes obtained as a result of crossing 1) *y^1^ v^1^; P{*y^+t7^.^7^ = *CaryP}attP40* and *y^1^ sc^∗^ v^1^; P{y^+t7^.^7^ v^+t1^.^8^* = *TriP. HMS01751}attP40*; 2) *y^1^ v^1^; P{y^+t7^.^7^* = *CaryP}attP2* and *y^1^ v^1^; P{y^+t7^.^7^ v^+t1^.^8^* = *TriP. JF01255}attP2*; 3) *w[1118]* and *w[1118]; P{w[*+ *mC]* = *UAS-sgg.A81T}MB2*; 4) *w[1118]* and *w[1118]; P{w* + *mC* = *UAS-sgg.Y214F}2*; 5) *w[1118]* and *w[1118]; P{w* + *mC* = *UAS-sgg.B}*MB5 females, respectively, with *P{GawB}D42* and *w[1118]; P{w* + *mC* = *Ddc-GAL4.L}Lmpt4.36* males to induce the expression of transgenic constructs in motor and dopaminergic neurons, respectively. ^∗∗∗^*P* < 0.001, as determined by the Kruskal-Wallis test.

The overexpression of *sgg-RB Y214F* in motor neurons decreased male lifespan and increased female lifespan by 14%; the overexpression of *sgg-RB A81T* in motor neurons decreased male lifespan but did not affect female lifespan (Supplementary Table S3, Figures 3A,B, 4A, and Supplementary Figure S3A). In a second independent experiment, all the results were reproduced; in females with overexpression of *sgg-RB Y214F* in motor neurons, lifespan was increased by 17% (Supplementary Table S3 and Figures 3C,D). The overexpression of normal *sgg-RB* in motor neurons decreased the lifespan in males, the effect being stronger than the effect of overexpression of mutant copies, as well as in females (Trostnikov et al., 2019; Figure 4A and Supplementary Figure S3A). To fully characterize the pattern of changes in lifespan caused by various alterations in *sgg* expression in motor neurons, we measured lifespans in males and females with strong and weak *sgg* knockdowns. We used the same two lines with transgenic constructs providing *sgg* knockdown as were used in Trostnikov et al. (2019). The level of decrease was expected to depend on the effectiveness of the knockdown, according to the manufacturer’s description^7^. The strong knockdown was lethal, the weak knockdown decreased male lifespan but did not affect female lifespan, just like *sgg-RB A81T* overexpression (Supplementary Table S3, Figure 4A and Supplementary Figure S3A). This result confirmed a proposed similarity in GSK3 activity associated with weak knockdown and *sgg-RB A81T* overexpression.

On the resulting scale of effects caused by impaired *sgg* expression in female motor neurons (Figure 4A), an increase in life expectancy corresponded to moderate *sgg* overexpression. Mean lifespan (16% average increase) and maximum lifespan (age of 90% mortality, 10% average increase) were significantly elevated (Supplementary Tables S2, S3). Survival curves were also fitted by Gompertz function; comparison of the Gompertz function parameters showed that in females with *sgg-RB Y214F* overexpression in motor neurons, the initial level of mortality was lower in both experiments, whereas the age-dependent mortality rate was similar to the control mortality rate (Supplementary Table S2). Thus, we concluded that *sgg-RB Y214F* overexpression in motor neurons had a positive impact on both early mortality and maximum lifespan.

Bearing in mind the advantages of how the results of the weak knockdown and *sgg-RB A81T* overexpression in the fat body reinforced each other, we sought to find yet another way to increase GSK3 activity to confirm the nature of the dependence of female lifespan on this parameter. Additional lines expressing transgenes that encode *sgg* have been reported (Bourouis, 2002), however, we decided to look for unrelated approaches and drew attention to indirect ways of influencing GSK3 activity.

GSK3 is a kinase that has a high basal activity and is regulated via inactivation by different upstream regulators. Among these regulators, enzymes of the InR/TOR, Wnt and cell polarity pathways play an important role in the nervous system (Hur and Zhou, 2010). For example, the partitioning defective 3 (PAR3)-PAR6-atypical protein kinase C (aPKC) complex, which is involved in the control of cell division, is thought to inactivate GSK3. The inactivation of the complex should lead to increased GSK3 activity compared to the normal level. Although the interaction between aPKC and GSK3 can be complex, as the latter was shown to directly phosphorylate the first (Colosimo et al., 2010), we attempted to determine whether and how the putative retention of GSK3 activity in the absence of aPKC affects the lifespan of males and females.

**FIGURE 5:**
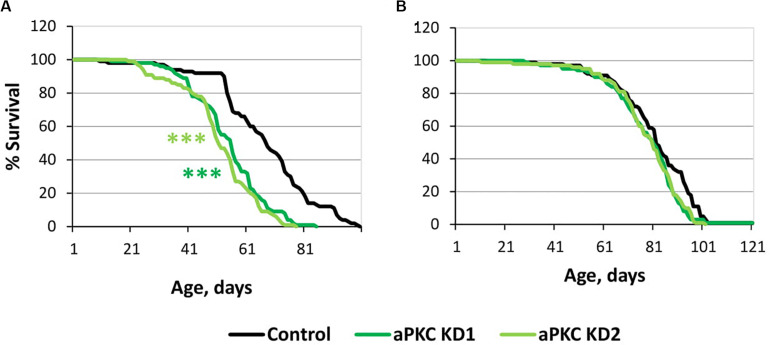
Effects of *aPKC* knockdown in motor neurons on male **(A)** and female **(B)** survival. Control, aPKC KD1, and aPKC KD2 denote hybrid genotypes obtained as a result of crossing *y[1] v[1]; P{y[* + *t7.7]* = *CaryP}attP40*, *y[1] sc[^∗^] v[1]; P{y[* + *t7.7] v[* + *t1.8]* = *TRiP.HMC06305}attP40* and *y[1] sc[^∗^] v[1]; P{y[* + *t7.7] v[* + *t1.8]* = *TRiP.HMS01689}attP40* females, respectively, with *w^∗^; P{GawB}*D42 males to induce the expression of transgenic constructs in motor neurons. ***P < 0.001, as determined by the Mann-Whitney test.

For this trial experiment, we decided to use two lines expressing transgenic constructs that provide RNA interference, namely, aPKC KD1 (*y[1] sc[^∗^] v[1]; P{y[* + *t7.7] v[* + *t1.8]* = *TRiP. HMC06305}attP40*) and aPKC KD2 (*y[1] sc[^∗^] v[1]; P{y[* + *t7.7] v[* + *t1.8]* = *TRiP. HMS01689}attP40), and a control (y[1] v[1]; P{y[* + *t7.7]* = *CaryP}attP40*) line without transgenic insertions obtained from the Bloomington Drosophila Stock Center (USA)^3^. Experiments with two different fly lines gave similar results. *aPKC* knockdowns in motor neurons decreased male lifespan by approximately 25% (Supplementary Table S4 and Figure 5A), while female lifespan was not affected (Supplementary Table S4 and Figure 5B). These results did not contradict our assumption considering the effects of aPKC on GSK3.

The overexpression of *sgg-RB Y214F* in dopaminergic neurons decreased male lifespan but did not affect female lifespan; the overexpression of *sgg-RB A81T* in dopaminergic neurons decreased male lifespan and increased female lifespan by 10% (Supplementary Table S3 and Figures 3E,F). In a second independent experiment, all the results were reproduced; in females with overexpression of *sgg-RB A81T* in dopaminergic neurons, lifespan was increased by 16% (Supplementary Table S3 and Figures 3G,H). The overexpression of normal *sgg-RB* in dopaminergic neurons was lethal in males and females (Trostnikov et al., 2019; Figure 4B) and Supplementary Figure S3B). To fully characterize the pattern of changes in lifespan caused by various alterations in *sgg* expression in dopaminergic neurons, we measured lifespans in males and females with strong and weak *sgg* knockdowns. The strong knockdown severely reduced male and female lifespans, the weak knockdown decreased male and female lifespans to a small degree (Supplementary Table S3, Figure 4B, and Supplementary Figure S3B). The patterns of effects in motoneurons and dopaminergic neurons were “shifted” relative to each other: in motoneurons, strong knockdown most critically affected lifespan, causing lethality, while in dopaminergic neurons strong overexpression lead to a lethal effect (Figure 4 and Supplementary Figure S3).

In accordance with this “shift,” on the resulting scale of effects caused by impaired *sgg* expression in female dopaminergic neurons (Figure 4), an increase in life expectancy corresponded to the dominant negative effect. Mean lifespan (13% average increase) and maximum lifespan (age of 90% mortality, 19% average increase) were significantly increased in both experiments (Supplementary Tables S2, S3). Survival curves were fitted by Gompertz function; comparison of the Gompertz function parameters showed that in females with *sgg-RB A91T* overexpression in dopaminergic neurons, the age-dependent mortality rate was 9 and 30% smaller in the first and in the second experiment, respectively, compared to controls (Supplementary Table S2). Thus, we concluded that *sgg-RB A81T* overexpression in dopaminergic neurons had a positive impact on maximum lifespan and, probably, on mortality rate.

Overall, our results confirmed that *sgg* misexpression in several tissues had a sex-specific impact on lifespan and aging. The fine-tuning of GSK3 activity allowed us to observe not only detrimental but also advantageous effects on lifespan. Data on the role of *sgg* expression in individual neurons in controlling lifespan deserved our greatest interest. Remarkably, the direction of the effect on lifespan was specifically determined by both the direction of the presumed change in the activity of GSK3 and the type of nerve cell. We were most interested in further analysis of GSK3-dependent cellular mechanisms underlying positive changes in lifespan.

### Overexpression of *sgg-RB Y214F* in Motor Neurons Affects Structural and Functional Properties of the Nervous System

Motor neurons are specialized cells of the nervous system that innervate muscles. Neuromuscular junctions (NMJs) are responsible for transforming neuronal signals into motor activity. In recent years, there has been considerable interest in the cellular and molecular bases of motor neuron performance because a number of diseases, such as ALS, sarcopenia and different types of spinal cord disturbances, have a strong correlation with abnormal motor neurons (Li et al., 1995; Drey et al., 2014; Peters et al., 2015). It was shown that changes normally observed with aging did not occur in the NMJs of motor neurons of flies with increased longevity (Liao et al., 2017), which indicated an association between the functional status of motor neurons and the aging process.

To better understand the association between increases in lifespan and alterations in motor neuron characteristics upon an implied moderate increase in GSK3 activity, we characterized the effects of *sgg-RB Y214F* overexpression in motor neurons in more detail.

**FIGURE 6:**
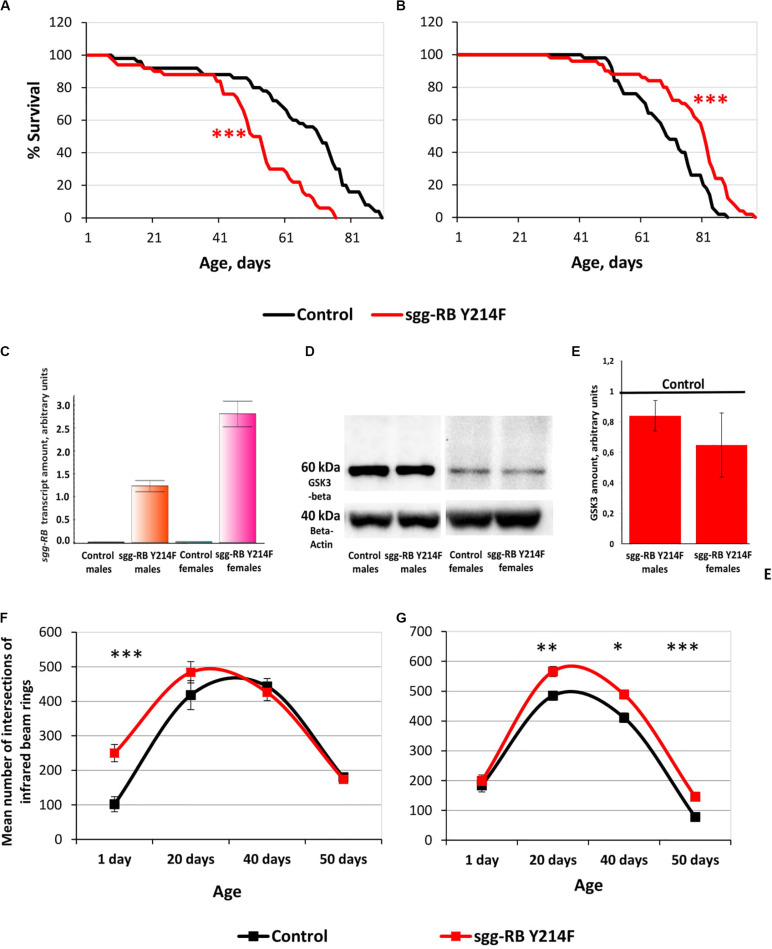
Effects of moderate changes in *sgg* expression in motor neurons on male **(A)** and female **(B)** survival and *sgg-RB* transcript levels **(C)**, GSK3 levels **(D,E)**, and locomotion in males **(F)** and females **(G)**. Control and sgg-RB Y214F denote hybrid genotypes obtained as a result of crossing *w[1118]* and *w[1118]; P{w* + *mC* = *UAS-sgg.Y214F}2* females, respectively, with *w^∗^; P{GawB}*D42 males to induce the expression of transgenic constructs in motor neurons. Typical results of real-time RT-qPCR **(C)** and Western blotting **(D)** and the quantification of GSK3 levels based on three biological replicates **(E)** are shown. *P < 0.05, **P < 0.01, and ***P < 0.001, as determined by the Mann-Whitney test **(A,B)** and Kruskal-Wallis test **(F,G)**.

The impact of *sgg-RB Y214F* overexpression in motor neurons on male and female lifespans was additionally evaluated in a third independent experiment, which was performed at the same time as the other experiments described in this section, to confirm that the effects on the lifespan remained the same. The new data perfectly replicated the previously obtained results (Supplementary Table S2 and Figures 6A,B).

Overexpression of additional copies of the gene is expected to increase the levels of RNA and, as a result, the protein encoded by this gene. Importantly, the levels of RNA and the protein should be the same when overexpressing both normal and mutant copies of the gene, if the mutation does not affect the level of expression, which is not the case in our study. To verify that the GAL4-UAS system worked, we assessed changes in the levels of *sgg-RB* transcript and GSK3 protein induced by *sgg-RB Y214F* overexpression in motor neurons using real-time RT-qPCR and Western blotting techniques, respectively. The increase in the level of *sgg-RB* transcript was confirmed in the male and female thorax, where motor neurons are located; typical results are illustrated in Figure 6C. Typical results of Western blotting are illustrated in Figure 6D, and the quantified results of three independent biological replicates are shown in Figure 6E. Western blot should detect both mutant GSK3 and the endogenously expressed normal GSK3. They are of the same size, and antibodies used recognize an epitope that the mutation does not affect. Surprisingly, in individuals with *sgg-RB Y214F* overexpression, an increase in the level of the GSK3 isoform that is roughly equivalent in size to the main Sgg-RB isoform was not confirmed. The significant effect was absent both in males and females (*P* = 0.4867, *P* = 0.1025, respectively, Kruskal-Wallis test). Considering that the mutant protein may possibly form dimmers (Fraser et al., 2002), we hypothesized that an increase in the amount of 120 kD protein would occur, but did not reveal it. Indeed, even if the mutant protein forms a certain number of dimers, they are not stable (Fraser et al., 2002), and monomers should dominate. Earlier, we demonstrated that the overexpression of the normal *sgg-RB* by the panneuronal driver caused an increase in the level of GSK3 protein in the brain (Trostnikov et al., 2019), however, the increase was not strong. It is likely that overexpression is somehow compensated for by cells to reduce the imbalance. In addition, motor neurons make up a small part of the mass of the thorax, and changes in the level of GSK3 in them can be masked by unaffected GSK3 levels in other cells. As a result, when analyzing proteins from the whole thorax, the effect of *sgg-RB Y214F* overexpression in motor neurons on the level of GSK3 may remain undetected. This explains why we might not see the expected effects at the protein level, even though the GAL4-UAS system did work. As mentioned above, the ability to directly compare GSK3 activity in motor neurons with and without *sgg-RB Y214F* overexpression is technically questionable. We, however, detected the effects of *sgg-RB Y214F* overexpression on lifespan, which indicated that there was a change in GSK3 activity in motor neurons; most likely, as anticipated (Bourouis, 2002), this change was a moderate increase. Bourouis (2002) showed that, with respect to a few phenotypes, *sgg-RB Y214F* overexpression demonstrated a dominant-negative effect, however, it should be kept in mind that, in our work, we clearly observed differences between the effects of *sgg-RB Y214F* and *sgg-RB A81T* overexpression on lifespan, and the latter transgene is considered by several authors (Bourouis, 2002; Franco et al., 2004; Franciscovich et al., 2008; Cuesto et al., 2015) to have a dominant-negative effect.

To assess the functional status of motor neurons in individuals with *sgg-RB Y214F* overexpression, we measured the locomotor activity of flies. In young 1–3 days old males with *sgg-RB Y214F* overexpression in motor neurons, locomotion was increased compared to that in control males; however, in 20, 40, and 50 days old males, this difference was not detected (Figure 6F). In both groups of males, locomotion reached its maximum at 20–40 days of age and then rapidly declined. Notably, the slope of the survival curves of both control males and males with *sgg-RB Y214F* overexpression became steeper at the age of 40–50 days, indicating an increase in the rate of aging (Figure 6A). Thus, as previously supposed (Ridgel and Ritzmann, 2005) and as we showed earlier in a study of the overexpression of normal sgg-RB in motor neurons (Trostnikov et al., 2019), locomotion is a reasonably good marker of aging. At the same time, in males, *sgg-RB Y214F* overexpression decreased lifespan but did not affect the locomotion of middle-aged or old individuals and even increased it in young individuals; this indicates that a decrease in lifespan is not necessarily associated with a decrease in locomotion and that the functional status of the nervous system necessary for ensuring motor functions was not impaired due to the presumed moderate increase in GSK3 activity.

In young 1–3 days old females with *sgg-RB Y214F* overexpression in motor neurons, locomotion was not changed compared to that in control females, but in 20, 40, and 50 days old females, locomotion was significantly increased compared to that in control females (Figure 6G). In both groups of females, locomotion reached its maximum at 20 days of age and became severely reduced at 50 days of age when the slope of both survival curves became steeper, indicating an increase in the rate of aging (Figure 6B). In females, *sgg-RB Y214F* overexpression increased both lifespan and locomotion, indicating that certain changes in the functional status of the nervous system can be accompanied by improved longevity.

In motor neurons, several cellular mechanisms, such as an increase in synaptic activity in NMJs and an increase in mitochondrial function, which determines the energy status of the cell, can underlie the observed increase in locomotion. To test these two possibilities, we evaluated the number of active synaptic zones and the number of mitochondria in larval NMJs, which are often used as a model system to study synaptic function in Drosophila (Ruiz-Cañada and Budnik, 2006). GSK3 is enriched in the presynaptic side of NMJs (Franco et al., 2004); in addition, mitochondria accumulate within nerve terminals to support neuronal firing, most notably through ATP production (Kann and Kovács, 2007). Therefore, we focused on studying the presynaptic zone.

**FIGURE 7:**
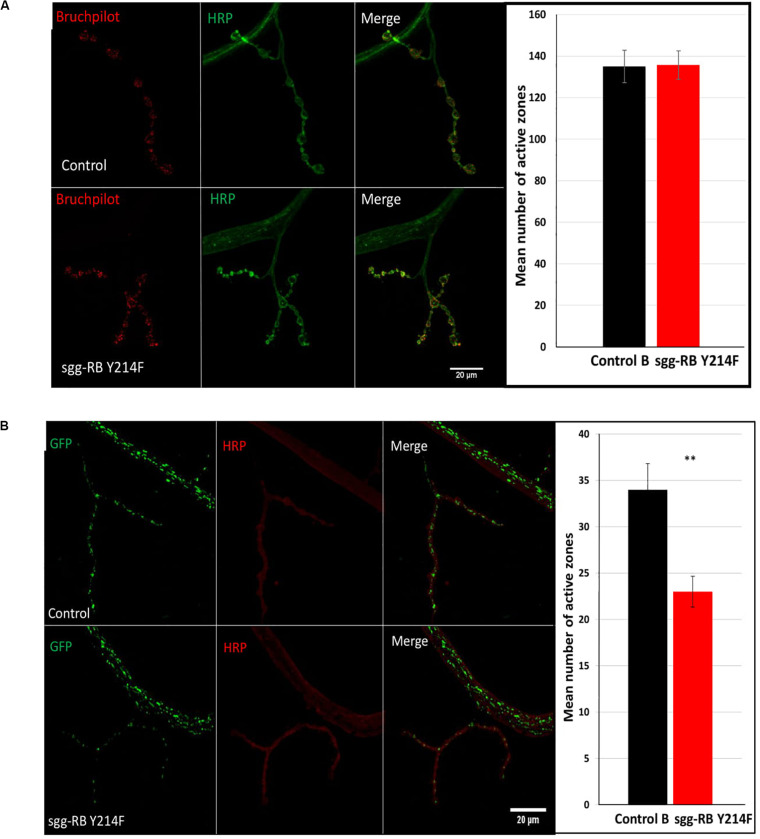
Effects of moderate changes in *sgg* expression in motor neurons on the number of active synaptic zones **(A)** and the number of mitochondrial clusters **(B)** in NMJs in females. Control and sgg-RB Y214F denote hybrid genotypes obtained as a result of crossing *w[1118]* and *w[1118]; P{w* + *mC* = *UAS-sgg.Y214F}2* females, respectively, with *w^∗^; P{GawB}*D42 **(A)** and *w[1118]; P{w* + *mC* = *UAS-mito-HA-GFP.AP}2/CyO; P{GawB}D42* **(B)** males to induce the expression of transgenic constructs in motor neurons. Representative confocal images of NMJs (muscle 4; hemisegment 3–4) stained for active synaptic zones (BRP, red) and neural membranes (HRP, green) **(A)** and for mitochondrial clusters (GFP, green) and neural membranes (HRP, red) **(B)**. Bar = 20 μm. The quantification of the number of active synaptic zones and mitochondrial clusters is shown. ^∗∗^*P* < 0.01, as determined by the Kruskal-Wallis test.

The number of active zones in larval NMJs was visualized by an antibody against Bruchpilot (BRP), a homolog of the vertebrate active zone protein ELKS (Wagh et al., 2006). The number of active synaptic zones in both male (data not shown) and female (Figure 7A) larvae with *sgg-RB Y214F* overexpression in motor neurons was not changed compared to that in the corresponding controls. It was previously shown that *sgg* overexpression affected the number of synaptic boutons (Franco et al., 2004), the number and size of synapses (Franciscovich et al., 2008; Cuesto et al., 2015), the integrity of presynaptic terminals and axons (Chiang et al., 2009) and synaptic activity (Trostnikov et al., 2019). However, moderate *sgg* overexpression appeared to not be sufficient to affect the synaptic activity of larval NMJs; accordingly, the increase in locomotion observed in females, most likely, cannot be explained by increased synaptic function.

It was previously shown that a decrease in lifespan was associated with a decrease in the number of active synaptic zones (Trostnikov et al., 2019), however, in flies with increased lifespan, the characteristics of active synaptic zones were not changed (Liao et al., 2017). Similarly, we also did not observe alterations in the number of active synaptic zones in females with increased longevity.

The functional integrity of postmitotic cells, such as neurons, critically depends on proper cellular energy supply. Mitochondrial dysfunction underlies numerous pathologies, including neurological pathologies (Kann and Kovács, 2007), and bearing in mind that *sgg* overexpression affects locomotion in a gender-dependent manner, it is interesting to note that sexual dimorphisms in mitochondria takes part in the sex specificity of important neurological disorders (Ventura-Clapier et al., 2017). We have already shown that strong *sgg* overexpression in motor neurons decreased the number of mitochondrial clusters in NMJs (Trostnikov et al., 2019), and this decrease was accompanied by an increase in locomotion. Here, we demonstrated that, in females with moderate *sgg* overexpression in motor neurons, the number of GFP-labeled mitochondrial clusters was also decreased (Figure 7B), while locomotion was also increased at the age of 20 days and older (Figure 6G). No changes in the number of mitochondrial clusters were revealed in males with moderate *sgg* overexpression in motor neurons (data not shown), in accordance with the absence of changes in locomotion at the age of 20 days and older (Figure 6F).

### Overexpression of *sgg-RB A81T* in Dopaminergic Neurons Affects Structural and Functional Properties of the Nervous System

Dopaminergic neurons are well known to be an important source of dopamine, and they play an essential role in multiple physiological and behavioral processes such as locomotion, cognition, mood, and stress. Dopamine-transmitting nerve cells have been studied for decades mainly due to their paramount importance in the development of Parkinson’s disease, the second most important age-related neurodegenerative disease (Cacabelos, 2017) and other brain pathologies (Chuhma et al., 2017; Nobili et al., 2017). There is a well-established correlation between age, dopaminergic neuron pathology and the development of neurodegenerative disorders (Collier et al., 2017).

To better understand the association between increases in lifespan and alterations in the properties of dopaminergic neurons upon an implied moderate decrease in GSK3 activity, we characterized the effects of *sgg-RB A81T* overexpression in dopaminergic neurons in more detail.

**FIGURE 8:**
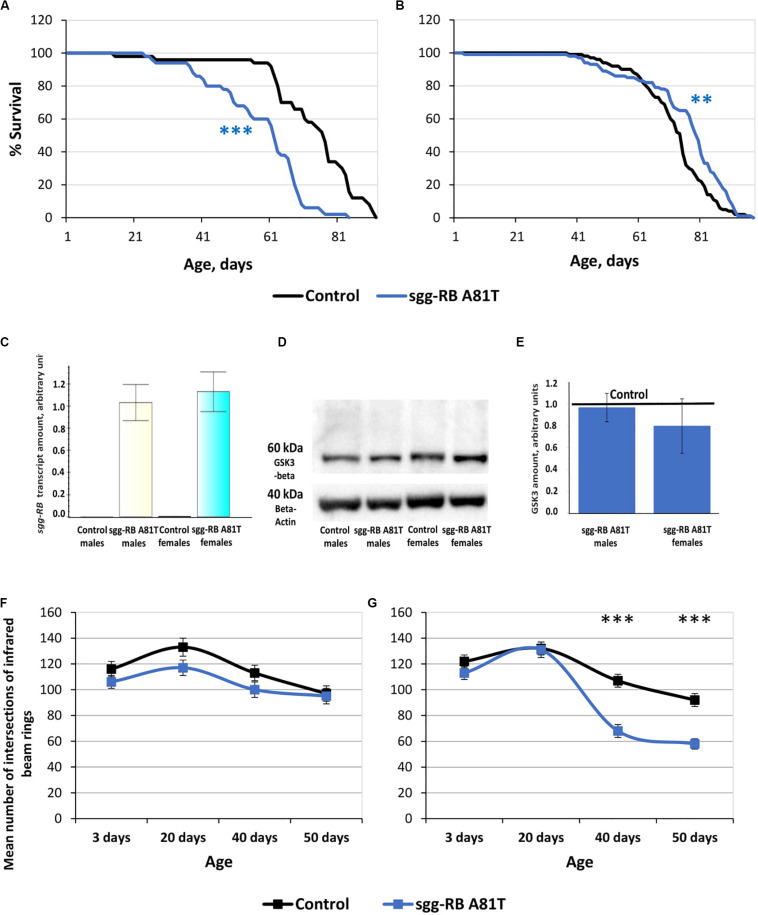
Effects of moderate changes in *sgg* expression in dopaminergic neurons on male **(A)** and female **(B)** survival and *sgg-RB* transcript levels **(C)**, GSK3 levels **(D,E)**, and locomotion in males **(F)** and females **(G)**. Control and sgg-RB A81T denote hybrid genotypes obtained as a result of crossing *w[1118]* and *w[1118]; P{w[*+ *mC]* = *UAS-sgg.A81T}MB2* females, respectively, with *w[1118]; P{w* + *mC* = *Ddc-GAL4.L}Lmpt4.36* males to induce the expression of transgenic constructs in dopaminergic neurons. Typical results of real-time RT-qPCR **(C)** and Western blotting **(D)** and the quantification of GSK3 levels based on four biological replicates **(E)** are shown. **P < 0.01, ***P < 0.001, as determined by the Mann-Whitney test **(A,B)** and Kruskal-Wallis test **(F,G)**.

The impact of *sgg-RB A81T* overexpression in dopaminergic neurons on male and female lifespans was additionally evaluated in a third independent experiment, which was performed at the same time as the experiments described below, to confirm that the effects on lifespan remained the same. The new data replicated the previously obtained results (Supplementary Table S2 and Figures 8A,B).

To verify that the GAL4-UAS system worked, we assessed the changes in the levels of *sgg-RB* transcript and GSK3 protein caused by *sgg-RB A81T* overexpression in dopaminergic neurons, we used real-time RT-qPCR and Western blotting techniques, respectively. The increase in the level of *sgg-RB* transcript was confirmed in male and female brains, where dopaminergic neurons are mainly located; typical results are illustrated in Figure 8C. Typical results of Western blotting are illustrated in Figure 8D, and the quantified results of four independent biological replicates are shown in Figure 8E. Similar to the results obtained in flies with *sgg-RB Y214F* overexpression in motor neurons, in individuals with *sgg-RB A81T* overexpression in dopaminergic neurons, an increase in the level of the GSK3 isoform Sgg-RB was not observed. The significant effect was absent both in males and females (*P* = 0.9999, *P* = 0.2186, respectively, Kruskal-Wallis test). It is worth mentioning that, although the dominant-negative effect of *sgg-RB A81T* overexpression has never been questioned (Bourouis, 2002; Franco et al., 2004; Franciscovich et al., 2008; Cuesto et al., 2015), the mechanism of this effect remains obscure, and it is not clear whether an increase in the GSK3 level due to *sgg-RB A81T* overexpression or a decrease in the GSK3 level due to compensation resulting from unknown cellular mechanisms should be expected. To add to the complexity, in both cases, the effects of overexpression in dopaminergic neurons might be masked by other neurons when analyzing whole brains.

To assess the functional status of dopaminergic neurons, we measured the locomotion of flies. It is well known that damage to dopaminergic neurons in Parkinson’s disease leads to impaired motor activity (Cacabelos, 2017). In males with *sgg-RB A81T* overexpression in dopaminergic neurons, locomotion was visually somewhat decreased at all ages, but the difference was never significant (Figure 8F). Thus, the functional status of the nervous system necessary for ensuring motor functions was not impaired due to the presumed moderate decrease in GSK3 activity in dopaminergic neurons. These data once again confirmed that a decrease in lifespan is not necessarily associated with a decrease in locomotion. In young 3 and 20 days old females with *sgg-RB A81T* overexpression in dopaminergic neurons, locomotion was not changed compared to that in control females, but in 40 and 50 days old females, locomotion was significantly decreased compared to that in control females (Figure 8G). This is consistent with the fact that GSK3 dysregulation plays an important role in the pathogenesis of Parkinson’s disease, a neurodegenerative movement disorder characterized by dopaminergic neuron pathology (Li et al., 2014; Golpich et al., 2015). However, in our model females, *sgg-RB A81T* overexpression decreased locomotion but increased lifespan, indicating that negative changes in the functional status of the nervous system can be accompanied by improved longevity.

**FIGURE 9:**
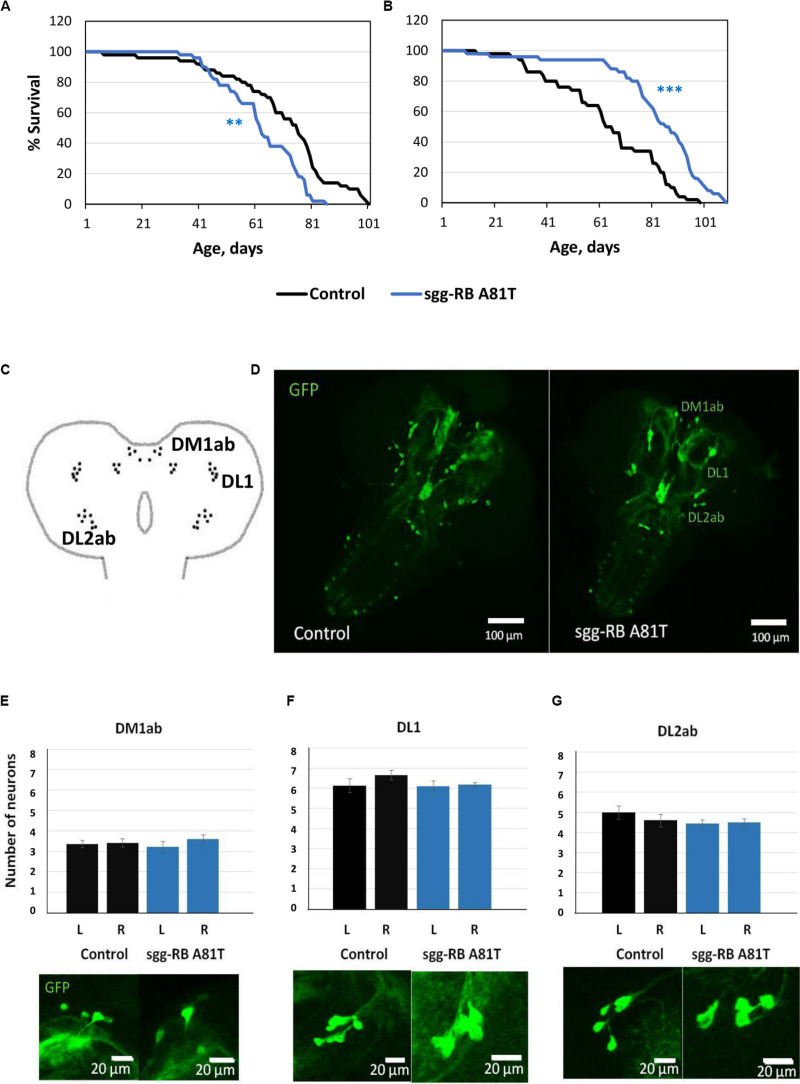
Effects of moderate changes in *sgg* expression in dopaminergic neurons on male **(A)** and female **(B)** survival and the number of dopaminergic neurons in the brains of female third instar larvae. Clusters of dopaminergic neurons in the central brain of a third instar larvae **(C)** are represented according to (Hartenstein et al., 2017). Control and sgg-RB A81T denote hybrid genotypes obtained as a result of crossing *w[1118]* and *w[1118]; P{w[*+ *mC]* = *UAS-sgg.A81T}MB2* females, respectively, with *w^∗^; P{w[*+ *mC]* = *UAS-mCD8:GFP.L}LL5/Cy; P{w* + *mC* = *ple-GAL4.F}3* males to induce the expression of transgenic constructs in dopaminergic neurons. A full description of the genotypes is given in “Materials and Methods” section. Representative confocal images of GFP-stained dopaminergic neurons **(D)** and the quantification of the number of neurons in different clusters based on 8–12 biological replicates **(E–G)** are shown. **P < 0.01, ***P < 0.001, as determined by the Mann-Whitney test.

In Parkinson’s disease, movement impairment is associated with the progressive and massive loss of dopaminergic neurons (Li et al., 2014; Golpich et al., 2015). We decided to examine how *sgg-RB A81T* overexpression affects dopaminergic neurons in females that exhibit increased locomotion and prolonged lifespan. To visualize dopaminergic neurons, we used the D11 driver line to induce the expression of transgenic constructs, including the one that is present in the line itself and encodes GFP, in dopaminergic neurons. In the D10 and D11 lines, different promoters are used to induce the expression of transgenic constructs. We examined whether *sgg-RB A81T* overexpression induced by the D11 driver in dopaminergic neurons affected male and female lifespans. The experiment reproduced the results previously obtained with the D10 driver (Supplementary Table S2 and Figures 9A,B), confirming an increase in female lifespan, which justified the use of the D11 driver for histochemical analyses.

**FIGURE 10:**
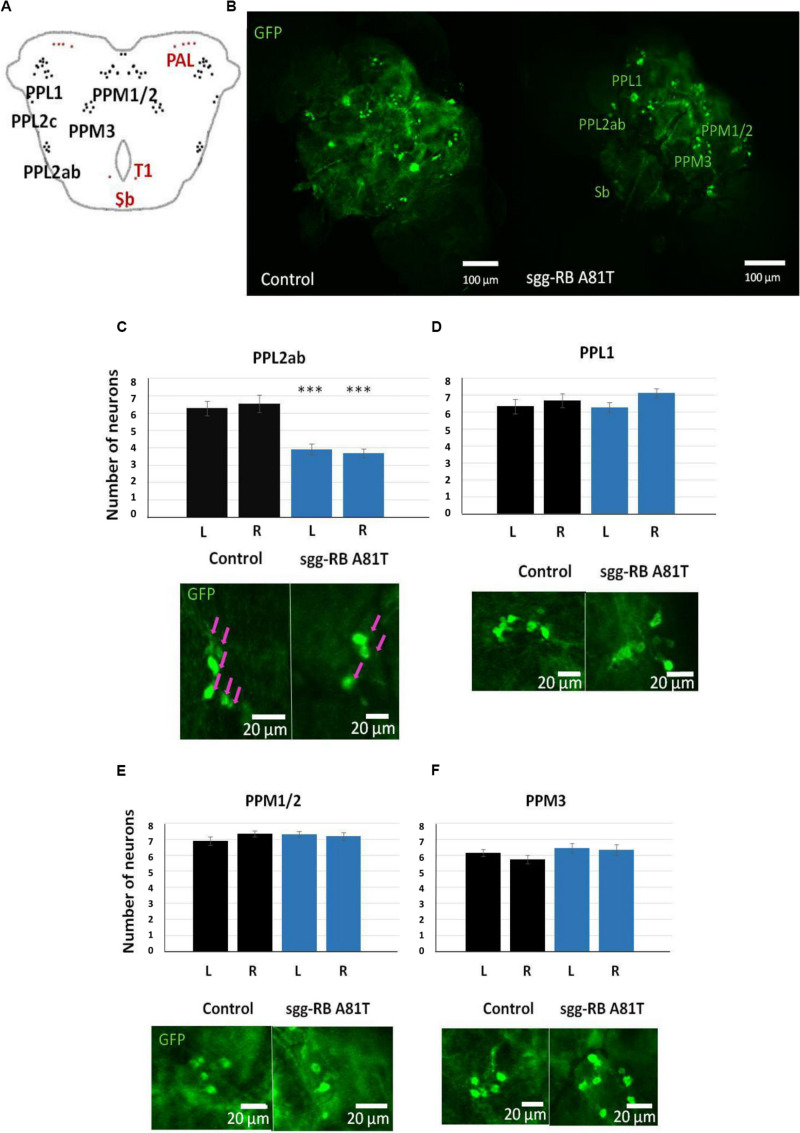
Effects of moderate changes in *sgg* expression in dopaminergic neurons on the number of dopaminergic neurons in the brains of 3–5 days old adult females. Clusters of dopaminergic neurons in the central brain of an adult fly **(A)** are represented according to (Xie et al., 2018). Clusters located in the anterior part of the brain are shown in red, and clusters located in the posterior part of the brain are shown in black. Control and sgg-RB A81T denote hybrid genotypes obtained as a result of crossing *w[1118]* and *w[1118]; P{w[*+ *mC]* = *UAS-sgg.A81T}MB2* females, respectively, with *w^∗^; P{w[*+ *mC]* = *UAS-mCD8:GFP.L}LL5/Cy; P{w* + *mC* = *ple-GAL4.F}3* males to induce the expression of transgenic constructs in dopaminergic neurons. Representative confocal images of GFP-stained dopaminergic neurons **(B)** and the quantification of the number of neurons in different clusters based on 10–16 biological replicates **(C–F)** are shown. ^∗∗∗^*P* < 0.001, as determined by the Kruskal-Wallis test. The red arrows indicate PPL2ab neurons.

**FIGURE 11:**
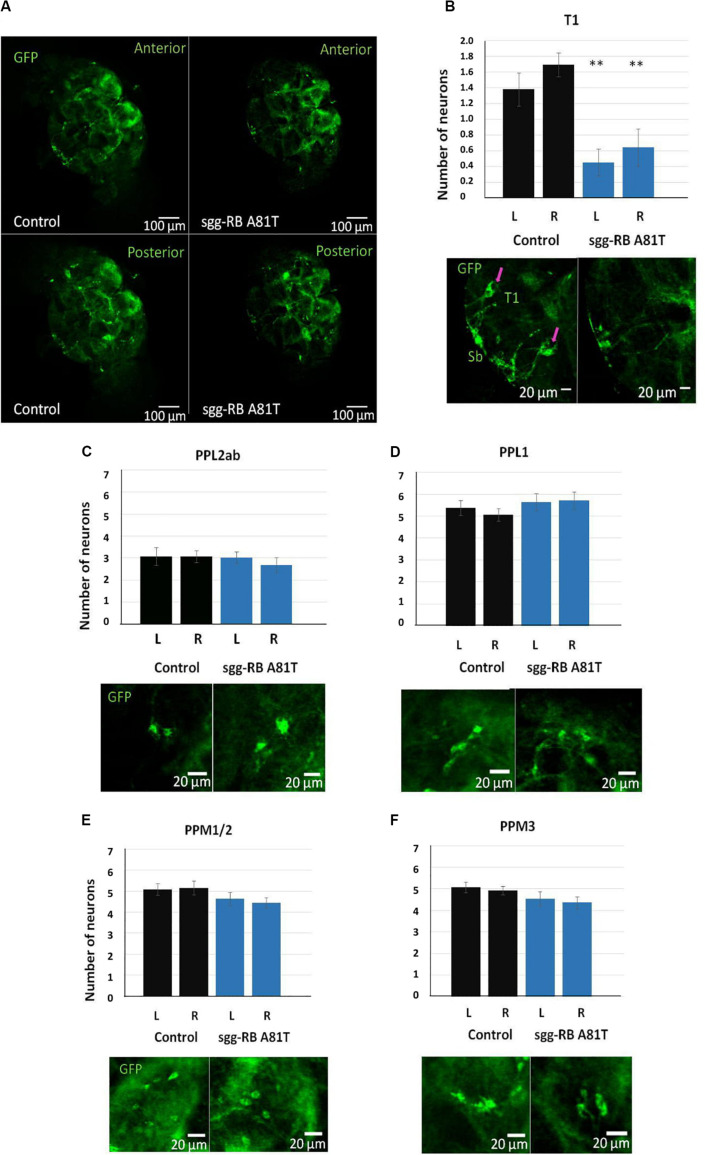
Effects of moderate changes in *sgg* expression in dopaminergic neurons on the number of dopaminergic neurons in the brains of 50 days old adult females. Control and sgg-RB A81T denote hybrid genotypes obtained as a result of crossing *w[1118]* and *w[1118]; P{w[*+ *mC]* = *UAS-sgg.A81T}MB2* females, respectively, with *w^∗^; P{w[*+ *mC]* = *UAS-mCD8:GFP.L}LL5/Cy; P{w* + *mC* = *ple-GAL4.F}3* males to induce the expression of transgenic constructs in dopaminergic neurons. Representative confocal images of GFP-stained dopaminergic neurons **(A)** and the quantification of the number of neurons in different clusters based on 9–13 biological replicates **(B–F)** are shown. ^∗∗^*P* < 0.01, as determined by the Kruskal-Wallis test. The red arrows indicate T1 neurons.

In the central brain of third instar larvae, as well as in the central brain of adult *D*. *melanogaster*, dopaminergic neurons are organized into a number of anatomically defined clusters (Figures 9C, 10A). We analyzed the numbers of neurons in most clusters, namely, DM1a/b, DL1, and DL2a/b in the larval brain; PAL, T1, and Sb in the anterior part of the adult brain; and PPL1, PPL2ab, PPL2c, PPM1/2, PPM3 in the posterior part of the adult brain. In several clusters (the DM2 cluster in the larval brain and the PAM cluster in the adult brain), we considered the estimates unreliable, and these clusters were excluded from consideration. At the larval stage, the number of neurons in the DM1a/b, DL1, and DL2a/b clusters in both the left and right brain hemispheres in females with *sgg-RB A81T* overexpression was not significantly different from the number of neurons in the same clusters in control females (Figures 9B–G). In young 3–5 days old females with *sgg-RB A81T* overexpression, the number of neurons in the PPL2a/b cluster in both the left and right brain hemispheres was significantly decreased compared to that in the controls (Figures 10B,C), however, in other clusters, differences were not revealed (Figures 10B,D–F). In old 50 days old females with *sgg-RB A81T* overexpression, the number of neurons in the T1 cluster in both the left and right brain hemispheres was significantly decreased compared to that in the controls (Figures 11A,B), however, in other clusters, differences were not revealed (Figures 11A,C–F). Overall, *sgg-RB A81T* overexpression decreased the number of dopaminergic neurons in certain clusters in females depending on age.

## Discussion

In general, to determine how the effects on lifespan depend on fine-tuning the level of GSK3 activity in different tissues, it would be helpful to be able to use independent methods to change GSK3 activity, to confirm the conclusions, especially those concerning improvements in lifespan. For example, the nature of the effects of dominant-negative mutations and RNAi knockdown on GSK3 activity is different, as the first affects protein function while the second affects transcript levels. Nevertheless, as mentioned above, the dominant-negative effects of the overexpression of *sgg-RB A81T* and weak *sgg* knockdown in the fat body on female lifespan largely confirmed each other. The presumed gradual decrease in GSK3 activity associated with the dominant-negative effects of *sgg-RB A81T* overexpression, weak knockdown and strong knockdown led to positive, nearly positive and negative changes in female lifespan, respectively. We believe that dominant-negative mutations (or loss-of-function mutations, if available) and RNAi knockdown can provide good opportunities for confirming the phenotypic effects of a decrease in gene/protein expression. Another approach that we just attempted to use is to study the effects of GSK3 partners (targets and regulators) on lifespan. Our data allowed us to hypothesize that the presumed gradual increase in GSK3 activity associated with strong *sgg* overexpression, *aPKC* knockdown and moderate *sgg* overexpression lead to negative changes, neither negative nor positive changes, and positive changes, respectively, in female lifespan. Of course, these findings do not allow us to fully understand how the two kinases interact with each other and how exactly modifications in the level of GSK3 activity in motor neurons might be responsible for gradual changes in lifespan, but they indicate a direction for future analyses. This approach could also allow a better understanding of the molecular mechanisms underlying GSK3 effects on lifespan.

Lifespan of individuals of different sexes varies in many species, including humans. The sex-specificity of lifespan can be based on systemic differences between sexes founded during the early development and sex determination process, which, later in life, causes sexual dimorphism in gene expression, metabolism and physiology. In this paper, positive effects on lifespan were revealed only in females. Though the molecular mechanisms that determine the differences in lifespan between the sexes remain largely unclear, some alterations in gene transcription and pathway regulation were shown to affect lifespan in a sex-specific manner (Tower, 2017; Garratt, 2019). For example, sex-specific effects on lifespan may be partly attributed to differences to Insulin and TOR signaling, while it is known that GSK3 is involved in these pathways. It was also demonstrated that female *D. melanogaster* showed greater lifespan extension with diet restriction due to the sexual dimorphism in gut pathology developed during aging (Regan et al., 2016). A widely accepted point of view is that the fundamental evolutionarily-conserved systemic regulation of aging by the reproductive system may account for the sex-specificity of lifespan control (Flatt, 2011). Several interesting observations were directly related to GSK3. GSK3-mediated cell survival was restricted to leukemic progenitors from female but not male patients because Receptor for Activated C Kinase 1 regulated GSK3 depending on patients’ gender (Bertrand et al., 2012). GSK3 activity is regulated by phosphorylation and dephosphorylation of Tyr216. A much larger amount of non-phosphorylated GSK3 was found in aged female mouse brains, compared to brains of male mouse (Krishnankutty et al., 2017). A reasonable assumption would be that in female brains GSK3 is overall less active than in male brains. If this is true for *D. melanogaster* as well, such a difference between males and females may affect the results of fine-tuning of GSK3 activity in different sexes.

The *D*. *melanogaster* fat body is an important tissue that participates in nutrient sensing, energy storage and the immune response. The activity of several key lifespan regulators [forkhead box protein (FOXO); target of rapamycin (TOR); tuberous sclerosis 2 (TSC2); and Sir2] in the fat body is essential for lifespan control (Giannakou et al., 2004; Hwangbo et al., 2004; Kapahi et al., 2004; Hoffmann et al., 2013). For example, the downregulation of TOR signaling in this tissue was shown to increase lifespan (Kapahi et al., 2004). GSK3 has an inhibitory effect on TOR signaling (Inoki et al., 2006); thus, a moderate decrease in GSK3 activity in the fat body would be expected to decrease lifespan, as was observed in males with *sgg-RB A81T* overexpression. It was also demonstrated that both the autonomous and non-autonomous activation of FOXO, the most important transcriptional effector of insulin-like and TOR signaling, in the adult fat body was shown to extend *D*. *melanogaster* lifespan (Giannakou et al., 2004; Hwangbo et al., 2004). Interestingly, in hepatoma cancer cells, GSK3 positively regulates the activity of FOXO and stimulates the FOXO-dependent activation of transcription, leading to cell proliferation (Huo et al., 2014). If GSK3 also positively regulates the activity of FOXO in the *D*. *melanogaster* fat body, a moderate decrease in GSK3 activity should lead to decreased lifespan, as was observed in males with *sgg-RB A81T* overexpression. However, the effects of GSK3 on insulin-like and TOR signaling cannot explain the increase in female lifespan.

Several mechanisms of the positive effect of a decrease in GSK3 activity on lifespan can be proposed. It was demonstrated that GSK3 is located in mitochondria, where it is more highly activated than in the cytosol (Bijur and Jope, 2003), and this mitochondrial GSK3 significantly promotes the production of reactive oxygen species (ROS) by inhibiting complex I, while GSK3 inhibition reverses this effect (King et al., 2008). The negative effects of substantially excessive ROS on lifespan are well known (Cui et al., 2012; Orr et al., 2013), and it is possible that, in our experiments, a moderate reduction in GSK3 activity in the fat body may have caused an increase in lifespan due to the mitigation of ROS production. The inhibition of GSK3 is also expected to enhance the production of heat shock proteins (HSPs) due to the attenuation of heat shock factor 1 (HSF-1) repression (Chu et al., 1996), which may also contribute to an increase in lifespan (Murshid et al., 2013). However, why these general mechanisms may only occur in the cells of the fat body remains obscure. Other putative mechanisms of prolonging lifespan through GSK3 inhibition can be deduced from Sutherland (2011).

In our experiments, alterations in GSK3 activity in a single group of nerve cells were sufficient to shift the rate of aging. The role of a small group of cells in lifespan control in *D*. *melanogaster* is well known. In the neurosecretory brain cluster, 14 cells homologous to mammalian pancreatic beta-cells produce insulin-like peptides (dilps) (Nässel and Vanden Broeck, 2016), and alterations in their levels have serious consequences for lifespan (Broughton et al., 2005, 2008; Post et al., 2018, 2019). Another impressive example of individual cells that are critically important for lifespan control is copper cells. This stomach-like cluster of specialized cells is localized in the middle midgut and plays an important role in age-related gut dysbiosis. It was demonstrated that the modification of the Jak/Stat pathway in these cells plays an important role in aging and affects lifespan (Li et al., 2016). Importantly, changes in the intestinal microbiome is tightly associated with the functionality of the nervous system: the microbiome-gut-brain axis is crucial to the development of some age-related neural pathologies (Sarkar and Banerjee, 2019).

An increase in GSK3 activity in the nerve cells is usually associated with the occurrence of serious neurological disorders (Ma, 2014). In particular, in amyotrophic lateral sclerosis, GSK3 expression is upregulated (Yang et al., 2008), and accordingly, glycogen synthesis is altered in association with impaired motor neuron function (Ferri and Coccurello, 2017; Li et al., 2019). At the same time, as glycogen synthesis leads to glucose storage, an excess of glycogen limits longevity, while the downregulation of the glycogen level is beneficial for lifespan (Gusarov et al., 2017; Seo et al., 2018). Thus, a moderate/slight increase in GSK3 activity, which can lead to a decrease in the level of glycogen, might increase lifespan, although there is no reason to attribute this effect to alterations in GSK3 activity in motor neurons. GSK3 has been proposed to restrain the oncogenic potential of several genes involved in the regulation of cell proliferation (Sutherland, 2011). In accordance with this, GSK3 activation promotes neuronal differentiation during neurogenesis (Hur and Zhou, 2010). In the nervous system, the coordinated phosphorylation of microtubule-associated proteins (MAP1, MAP2, and tau) by GSK3 is a major mechanism of the regulation of microtubule stability in neurons (Sutherland, 2011). It is possible that, in our experiments, a moderate elevation in GSK3 activity in motor neurons was advantageous for the well-being of the neurons. It remains to be elucidated how this might be connected to increased lifespan.

As a result of *sgg* overexpression in motor neurons, an increase in locomotion appeared to be associated with a decreased number of mitochondrial clusters in the presynaptic zone. This was a somewhat unexpected result, as mitochondrial loss is usually associated with negative effects on the functionality of the nervous system. Indeed, it was demonstrated that the loss of synaptic mitochondria is an early pathological change that might indicate future neurodegeneration (Zhu et al., 2013). In a transgenic *Drosophila* model of Alzheimer’s disease, mitochondrial mislocalization, i.e., a reduction in the number of mitochondria in axons contributes to neuronal dysfunction (Iijima-Ando et al., 2009). Earlier, we suggested (Trostnikov et al., 2019) that GSK3, which is involved in the control of mitochondrial movement (Morfini et al., 2002; Chen et al., 2007, 2008) and directly regulates dynein (Gao et al., 2015), providing anterograde mitochondrial trafficking, might be responsible for the distribution of mitochondria along axons. However, it remains obscure why mitochondrial deficiency in axonal terminals might be favorable for locomotion.

Many neurodegenerative diseases are characterized by the loss of neurons. In particular, Parkinson’s disease is characterized by the progressive and massive loss of dopaminergic neurons by neuronal apoptosis. It is generally accepted that GSK3 has a major role in neuronal apoptosis (Li et al., 2014; Maurer et al., 2014; Golpich et al., 2015) as well as a crucial role in oxidative stress in the loss of dopaminergic neurons (Drechsel and Patel, 2008). Interestingly, in cell culture under cell death-inducing conditions, the number of viable dopaminergic cells was increased following GSK3 inhibition and the subsequent activation of NRF2, which regulates responses to multiple stressors (Armagan et al., 2019). In mammals, reduced NRF2 expression was shown to mediate the decline in neural stem cell function (Corenblum et al., 2016; Robledinos-Antón et al., 2017), which could potentially lead to a decrease in the number of neurons. Given these data, somewhat unexpectedly, a moderate depletion of GSK3 caused a decrease in the number of particular (PPL2a/b) dopaminergic neurons in young flies in our model system. In larvae, the number of progenitors of these neurons (DL2a/b, Hartenstein et al., 2017) did not differ from the norm. It is known that, in larvae, neuroblasts enter a second phase of asymmetric divisions that give rise to some adult-specific secondary neurons (Hartenstein et al., 2017). All dopaminergic neurons, however, are primary neurons and are formed during the first embryonic phase of asymmetric divisions of neuroblasts, however, tyrosine hydroxylase expression in these neurons is initiated only at different larval stages, and some of them cannot be stained using the D11 driver and thus become visible until metamorphosis (Hartenstein et al., 2017). GSK3 is one of the key components ensuring asymmetric division (Colosimo et al., 2010), and GSK3 deregulation may well cause alterations in the formation of neurons. However, it is not clear why this effect is specific for the PPL2a/b cluster and, specifically, to neurons that are D11-negative in larvae. In old flies with GSK3 depletion, the number of these neurons returns to normal, indicating possibly improved survival. It was shown that GSK3 inactivation protected cells from mitochondria-mediated intrinsic cell apoptosis, which has been implicated in the pathogenesis of Parkinson’s disease, indicating its regulatory role in the mitochondrial cell death pathway (Li et al., 2014). This might be one of the possible mechanisms that allows better survival of dopaminergic neurons in old flies with GSK3 depletion due to the dominant-negative effect of *sgg-RB A81T* overexpression. The study of mitochondrial functions and mitochondria-mediated apoptosis in our model can shed light on this issue.

At first, it was not obvious how the very restricted and specific changes in the number of neurons could be associated with a decrease in female locomotion, and the association of the changes with an increase in female survival was even less obvious. However, the inactivation of neurons from the PPL1 cluster was shown to cause a slight deficit in movement in males, while males with inactivated T1 or PPM3 neurons were not different from controls (Alekseyenko et al., 2013). At the same time, both the activation and inactivation of T1 or PPM3 neurons resulted in increased aggression. These observations suggest that a small number of dopaminergic neurons can have substantial effects on complex traits. Currently, there is intense interest in dissecting the neural circuits in which dopaminergic neurons are involved, as they regulate a variety of vitally important processes; for this purpose, a genetic toolkit that allows the manipulation of small groups of dopaminergic neurons was developed (Xie et al., 2018). Future analyses should examine the putative role of PPL2a/b and T1 neurons in the control of locomotion and lifespan.

To search for the possible molecular mechanisms of the increase in lifespan due to the dominant-negative effect of *sgg-RB A81T* overexpression in dopaminergic neurons, we drew attention to nuclear factor erythroid 2-related factor 2 (NRF2), a transcription factor that regulates responses to multiple stressors. In particular, NRF2 mediates neuronal damage by modulating the expression of antioxidant enzymes (Tavakkoli et al., 2019). The activation of NRF2 due to suppression of the NRF2 repressor Keap1 extended the lifespan of *D*. *melanogaster* (Sykiotis and Bohmann, 2008; Spiers et al., 2019), and both Keap1 suppression and the constitutive overexpression of NRF2 positively modified neuronal function (Spiers et al., 2019). Another upstream regulator of NRF2 is GSK3, which also negatively controls NRF2 activity through phosphorylation and nuclear exclusion (Salazar et al., 2006; Jain and Jaiswal, 2007; Rada et al., 2011). The activation of NRF2 by reducing GSK3 activity can positively affect lifespan (Karim et al., 2015; Castillo-Quan et al., 2016). A plausible suggestion is that, in our experiments, a moderate reduction in GSK3 activity in dopaminergic neurons may have caused an increase in lifespan due to the weakened inhibition of NRF2 activity. It is possible that NRF2 can also mediate the effects of GSK3 depletion in the fat body, and *vice versa*, the general mechanisms mentioned above in the discussion of the effects found in the fat body can also explain the effects of GSK3 depletion in dopaminergic neurons on lifespan, especially because neuronal cells are particularly vulnerable to the levels of HSP and HSF-1 (Tonkiss and Calderwood, 2005).

## Conclusion

GSK3 is a ubiquitous enzyme present in the cytosol, nuclei and mitochondria (Bijur and Jope, 2003) and is involved in a wide variety of biological processes (McCubrey et al., 2016; Patel and Woodgett, 2017). More than 100 proteins are thought to be direct GSK3 targets; GSK3 phosphorylation affects protein activity, localization and stability, acting mainly on protein-protein interactions. Given this complexity, the question of how the enzyme is regulated so that it can participate in so many diverse processes, in particular, how the activity of the enzyme is distributed between different regulatory cascades and metabolic pathways, arises. It was suggested that, within a cell, small amounts of the enzyme can be distributed between different subcellular domains and in such a way are coupled to distinct signaling pathways (Patel and Woodgett, 2017). However, this does not elucidate the molecular mechanisms of the cellular specificity of GSK3 function when the same change in GSK3 activity in different types of cells leads to opposite cellular and phenotypic effects. For example, in this paper, we demonstrated that both an increase in GSK3 activity and a decrease in GSK3 activity can increase or decrease lifespan depending on the cell type. Another example is that GSK3 depletion in all dopaminergic neurons affects the number of neurons in only certain clusters. We also demonstrated that GSK3 deregulation in the cells of the fat body and motor and dopaminergic neurons caused an increase in lifespan and suggested possible molecular mechanisms underlying these effects, however, these suggestions do not explain why only females experienced improved longevity. This finding indicates that, in addition to explaining GSK3 allocation to different regulatory cascades within a cell through intracellular compartmentalization, we should determine and prove the mechanisms underlying the cellular and sexual specificity of GSK3 function. By raising questions about the cellular and sexual specificities of the effects of GSK3 and by providing model systems to study them, our work suggests the prospects for further research. Analysis of the molecular and cellular mechanisms that provide cellular and sexual specificity of the positive effects of GSK3 misexpression on survival is planned for the future.

It is currently well recognized that, in humans, it is crucial that an increase in lifespan is accompanied by an increase in healthspan, the functional and disease-free period of life (Hansen and Kennedy, 2016). In invertebrate models, such as *D*. *melanogaster*, the definition of healthspan is not obvious. However, locomotor activity is often regarded as a measure of healthspan (Hansen and Kennedy, 2016). We demonstrated that both an increase and a decrease in locomotion can accompany a prolonged lifespan. In both cases, however, the structural properties of the nervous system were seemingly impaired; the number of mitochondria in NMJs (in the first case) and the number of dopaminergic neurons (in the second case) were reduced. It remains an open question whether healthspan was considered impaired in our long-living flies with reduced locomotor activity. The effect was weak and might represent only a slight decrease in metabolism that did not affect quality of life and perhaps made it more relaxed. Of course, healthspan cannot be characterized by one trait/metric but requires a combination of measurements. A deeper analysis of the possible characteristics of healthspan in invertebrate models is an important undertaking for the future and is essential for drug development and testing.

## Data Availability Statement

All datasets generated for this study are included in the article/Supplementary Material.

## Author Contributions

MT, EV, and NR designed the experiments, collected, analyzed, and interpreted data, critically revised, edited the manuscript, and acquired funds. AK designed the experiments, analyzed and interpreted data, critically revised, and edited the manuscript. SB collected and interpreted the data, critically revised, and edited the manuscript. EP created the concept for the work, designed the experiments, analyzed and interpreted data, supervised the study, and drafted and critically revised the manuscript. All authors approved the version to be published.

## Conflict of Interest

The authors declare that the research was conducted in the absence of any commercial or financial relationships that could be construed as a potential conflict of interest. The reviewer EP declared a shared affiliation, with no collaboration, with several of the authors, MT, EV, AK, SB, NR, and EP to the handling editor at the time of review.

## Footnotes

1 https://flybase.org

2 http://flybase.org/reports/FBgn0003371

3 http://flystocks.bio.indiana.edu/

4 http://flystocks.bio.indiana.edu/Browse/TRiPtb.htm

5 http://rsb.info.nih.gov/ij/index.html

6 http://flybase.org/reports/FBti0002575.html

7 https://bdsc.indiana.edu/stocks/rnai/rnai_n_z.html
