# Supplementary material for: Modulated Expression of the Protein Kinase GSK3 in Motor and Dopaminergic Neurons Increases Female Lifespan in Drosophila melanogaster
Source: Front Genet. 2020 Jun 30;11:668. doi: 10.3389/fgene.2020.00668 (PMC7339944; doi:10.3389/fgene.2020.00668)
Supplement: Supplementary file 1 [file Image_1.pdf]

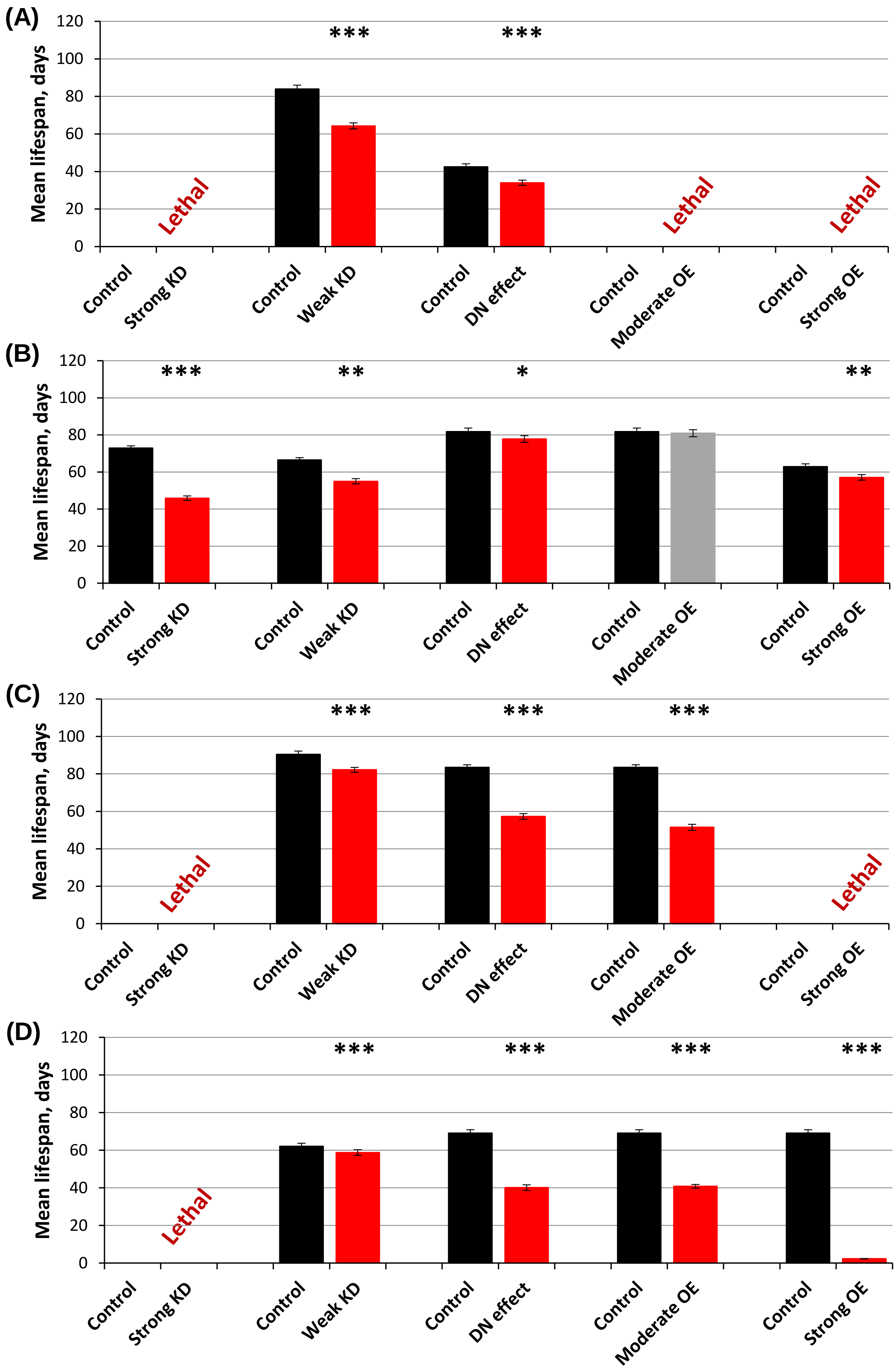
(A)
(B)
(C)
(D)
Mean lifespan, days
0
20
40
60
80
100
120
***
**
*
Lethal
Control
Strong KD
Weak KD
DN effect
Moderate OE
Strong OE

Fig. S1 Effects of multidirectional changes in *sgg* expression in embryos **(A)**, muscle **(B)**, the fat body **(C)** and the nervous systemc **(D)** on the mean lifespan of males. Control and Strong knockdown (KD); Control and Weak KD; Control and Dominant negative (DN) effect; Control and Moderate overexpression (OE); Control and Strong OE denote hybrid genotypes obtained as a result of crossing 1) *y^1 v^1; P{*$y^{+t7.7}$*=CaryP}attP40* and *y^1 sc* v^1; P{$y^{+t7.7}$ $v^{+t1.8}$=TriP. HMS01751}attP40*; 2) *y^1 v^1; P{$y^{+t7.7}$=CaryP}attP2* and *y^1 v^1; P{$y^{+t7.7}$ $v^{+t1.8}$=TriP. JF01255}attP2*; 3) *w[1118]* and *w[1118]; P{w[+mC]=UAS-sgg.A81T}MB2*; 4) *w[1118]* and *w[1118]; P{w+mC=UAS-sgg.Y214F}2*; 5) *w[1118]* and *w[1118]; P{w+mC=UAS-sgg.B}*MB5 females, respectively, with *y[1] w*; P{w+mW.hs=en2.4-GAL4}e22c; P{w+mC=tGPH}4/TM3, Ser[1]*, *P{w+mC=UAS-Dcr-2.D}1, w[1118]; P{w+mC=GAL4-Mef2.R}R1, w*; P{w+mC=ppl-GAL4.P}2* and *P{w+mW.hs=GawB}elavC155 w[1118]; P{w+mC=UAS-Dcr-2.D}2* males to induce the expression of transgenic constructs in embryos, muscle, the fat body and the nervous system, respectively. * denotes P < 0.05, ** denotes P < 0.01, and *** denotes P < 0.001, as determined by the Kruskal-Wallis test. Black asterisks denote differences between knockdowns and corresponding controls or overexpressions and corresponding controls; red asterisks denote differences between *sgg-RB* overexpression and *sgg-RB Y214F* or *sgg-RB A81T* overexpression. * P < 0.05, ** P < 0.01, and *** P < 0.001, as determined by the Kruskal-Wallis test.
