# Supplementary material for: Modulated Expression of the Protein Kinase GSK3 in Motor and Dopaminergic Neurons Increases Female Lifespan in Drosophila melanogaster
Source: Front Genet. 2020 Jun 30;11:668. doi: 10.3389/fgene.2020.00668 (PMC7339944; doi:10.3389/fgene.2020.00668)
Supplement: Supplementary file 2 [file Image_2.pdf]

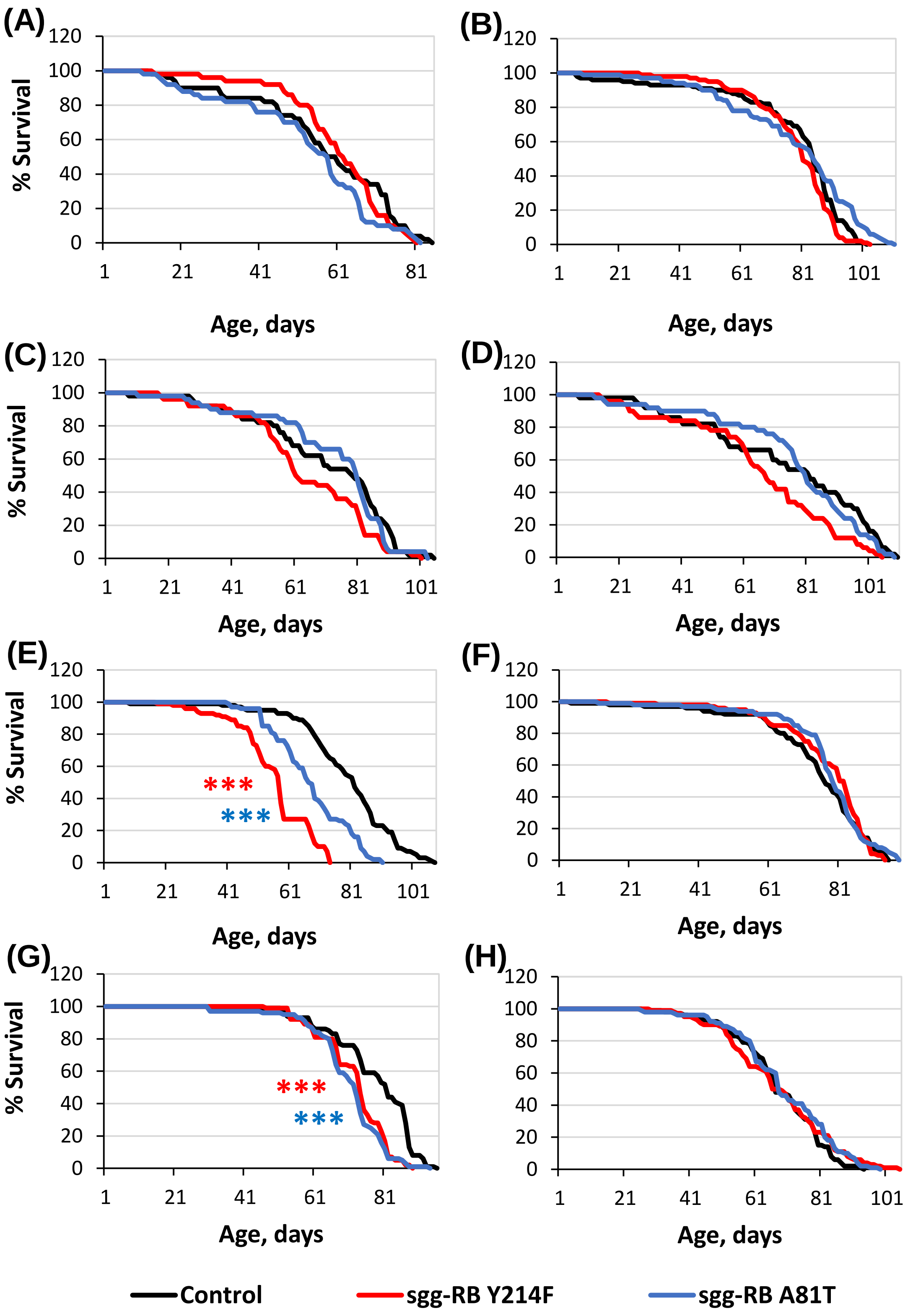

Fig. S2 Effects of moderate changes in *sgg* expression in peptidergic **(A)**, **(B)**, cholinergic **(C)**, **(D)**, glutamatergic **(E)**, **(F)** and GABAergic **(G)**, **(H)** neurons on male **(A)**, **(C)**, **(E)**, **(G)** and female **(B)**, **(D)**, **(F)**, **(H)** survival. Control, sgg-RB Y214F, and sgg-RB A81T denote hybrid genotypes obtained as a result of crossing *w[1118]*, *w[1118]; P{w+mC=UAS-sgg.Y214F}2* or *w[1118]; P{w[+mC]=UAS-sgg.A81T}MB2* females, respectively, with D5 (*w*; P{w+mW.hs=GawB}386Y*), D6 (*w*; P{w+mC=ChAT-GAL4.7.4}19B*), D7 (*w[1118]; P{w+mW.hs=GawB}VGlutOK371*), and D8 (*P{w+mC=Gad1-GAL4.3.098}2/CyO*) males to induce the expression of transgenic constructs in peptidergic, cholinergic, glutamatergic and GABAergic neurons, respectively. *** P < 0.001, as determined by the Mann-Whitney test.
