# Supplementary material for: Modulated Expression of the Protein Kinase GSK3 in Motor and Dopaminergic Neurons Increases Female Lifespan in Drosophila melanogaster
Source: Front Genet. 2020 Jun 30;11:668. doi: 10.3389/fgene.2020.00668 (PMC7339944; doi:10.3389/fgene.2020.00668)
Supplement: Supplementary file 3 [file Image_3.pdf]

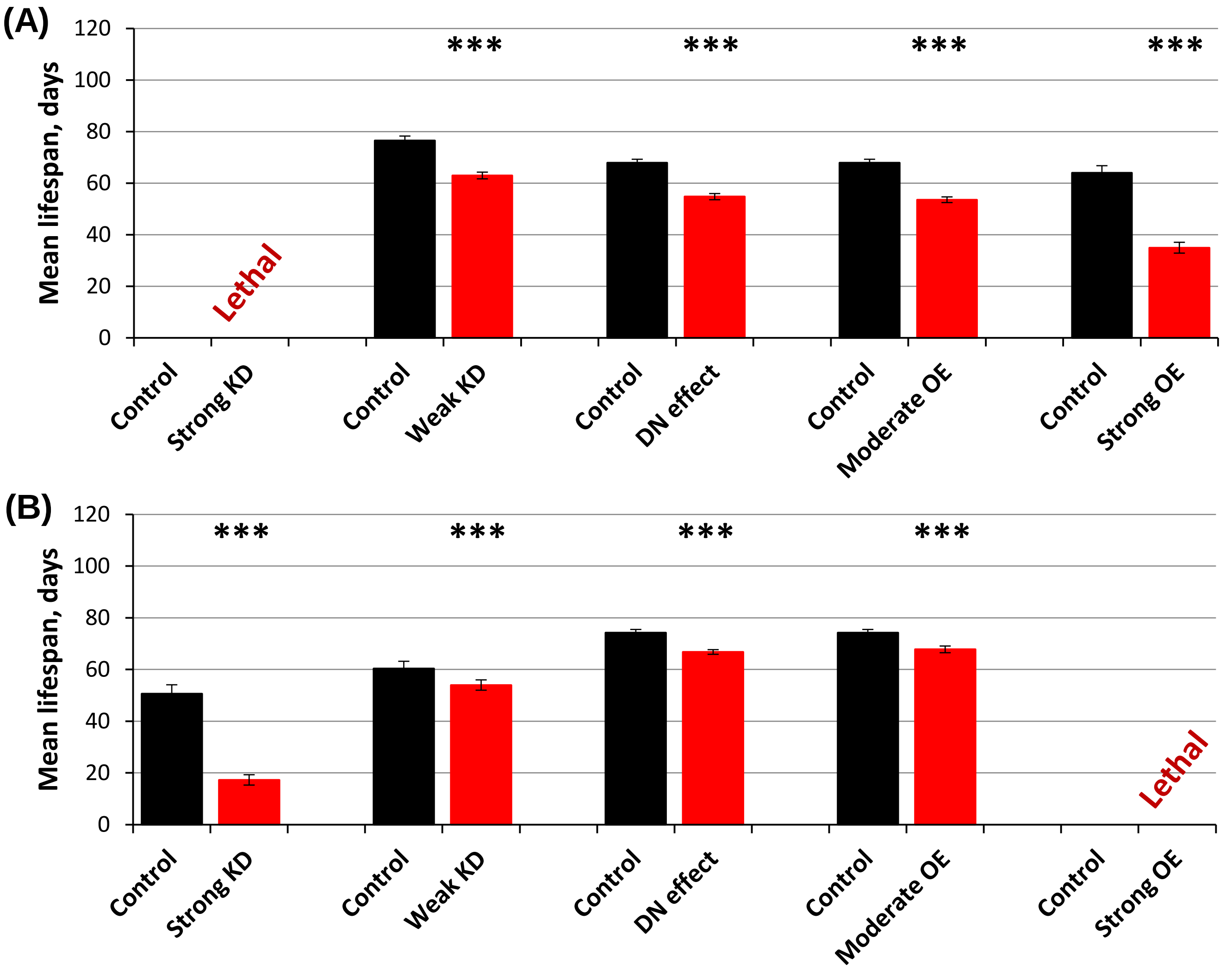

Fig. S3 Effects of multidirectional changes in *sgg* expression in motor **(A)** and dopaminergic **(B)** neurons on the mean lifespan of males. Control and Strong knockdown (KD); Control and Weak KD; Control and Dominant negative (DN) effect; Control and Moderate overexpression (OE); Control and Strong OE denote hybrid genotypes obtained as a result of crossing 1) y^1 v^1; *P{*$y^{+t7.7}$*=CaryP}attP40* and y^1 *sc** v^1; *P{*$y^{+t7.7}$ $v^{+t1.8}$*=TriP. HMS01751}attP40*; 2) y^1 v^1; *P{*$y^{+t7.7}$*=CaryP}attP2* and y^1 v^1; *P{*$y^{+t7.7}$ $v^{+t1.8}$*=TriP. JF01255}attP2*; 3) *w[1118]* and *w[1118]; P{w[+mC]=UAS-sgg.A81T}MB2*; 4) *w[1118]* and *w[1118]; P{w+mC=UAS-sgg.Y214F}2*; 5) *w[1118]* and *w[1118]; P{w+mC=UAS-sgg.B}*MB5 females, respectively, with *P{GawB}D42* and *w[1118]; P{w+mC=Ddc-GAL4.L}Lmpt4.36* males to induce the expression of transgenic constructs in motor and dopaminergic neurons, respectively. *** P < 0.001, as determined by the Kruskal-Wallis test.
