# Supplementary material for: Modulated Expression of the Protein Kinase GSK3 in Motor and Dopaminergic Neurons Increases Female Lifespan in Drosophila melanogaster
Source: Front Genet. 2020 Jun 30;11:668. doi: 10.3389/fgene.2020.00668 (PMC7339944; doi:10.3389/fgene.2020.00668)
Supplement: Supplementary file 4 [file Table_1.DOCX]

Table S1. Distributive statistics of the lifespan of transgenic flies with mutant *shaggy-RB* overexpresion in different tissues.

| Effects | Sex | Genotype | N | Mean | Median | Minimum | Maximum | Lower Quartile | Upper Quartile | Percentile 10 | Percentile 90 | Variance | Standard Deviation | Standard Error | P values for comparisons with control genotype | |
| --- | --- | --- | --- | --- | --- | --- | --- | --- | --- | --- | --- | --- | --- | --- | --- | --- |
|  |  |  |  |  |  |  |  |  |  |  |  |  |  |  | Mann-Whitney Test | Kolmogorov-Smirnov Test |
| ***sgg-RB Y214F*** overexpression in **embryos** | ♂ | Control | 100 | Not analyzed | | | | | | | | | | | | |
|  |  | Mutant | 100 | Lethal | | | | | | | | | | | | |
|  | ♀ | Control | 100 | Not analyzed | | | | | | | | | | | | |
|  |  | Mutant | 100 | Lethal | | | | | | | | | | | | |
| ***sgg-RB A81T*** overexpression in **embryos** | ♂ | Control | 100 | 42.5 | 45.0 | 7.0 | 68.0 | 35.5 | 53.0 | 18.0 | 61.0 | 254.6 | 16.0 | 1.6 |  |  |
|  |  | Mutant | 100 | 34.0 | 33.0 | 4.0 | 67.0 | 25.0 | 42.5 | 14.0 | 52.5 | 200.7 | 14.2 | 1.4 | **P < 0.0001** | **P < 0.001** |
|  | ♀ | Control | 100 | 60.1 | 63.0 | 6.0 | 87.0 | 51.5 | 70.5 | 42.5 | 77.5 | 248.8 | 15.8 | 1.6 |  |  |
|  |  | Mutant | 100 | 52.0 | 53.5 | 5.0 | 86.0 | 38.5 | 68.0 | 27.0 | 76.0 | 358.3 | 18.9 | 1.9 | **P = 0.0021** | **P < 0.005** |
| ***sgg-RB Y214F*** overexpression in **muscles** | ♂ | Control | 100 | 81.8 | 85.5 | 11.0 | 108.0 | 73.5 | 93.5 | 64.0 | 101.0 | 365.7 | 19.1 | 1.9 |  |  |
|  |  | Mutant | 100 | 80.9 | 87.5 | 9.0 | 105.0 | 70.5 | 95.0 | 54.5 | 99.0 | 377.7 | 19.4 | 1.9 | P = 0.9562 | P > 0.10 |
|  | ♀ | Control | 100 | 82.0 | 85.5 | 16.0 | 110.0 | 77.0 | 92.0 | 64.5 | 99.0 | 325.9 | 18.1 | 1.8 |  |  |
|  |  | Mutant | 100 | 80.6 | 85.0 | 9.0 | 110.0 | 77.0 | 96.0 | 39.5 | 104.0 | 572.3 | 23.9 | 2.4 | P = 0.5252 | P < 0.10 |
| ***sgg-RB A81T*** overexpression in **muscles** | ♂ | Control | 100 | 81.8 | 85.5 | 11.0 | 108.0 | 73.5 | 93.5 | 64.0 | 101.0 | 365.7 | 19.1 | 1.9 |  |  |
|  |  | Mutant | 100 | 77.8 | 81.0 | 6.0 | 111.0 | 70.0 | 88.0 | 63.5 | 95.0 | 317.7 | 17.8 | 1.8 | **P = 0.0179** | **P < 0.05** |
|  | ♀ | Control | 100 | 82.0 | 85.5 | 16.0 | 110.0 | 77.0 | 92.0 | 64.5 | 99.0 | 325.9 | 18.1 | 1.8 |  |  |
|  |  | Mutant | 100 | 86.5 | 92.0 | 13.0 | 114.0 | 79.0 | 100.0 | 64.0 | 105.0 | 371.2 | 19.3 | 1.9 | **P = 0.0079** | **P < 0.001** |
| ***sgg-RB Y214F*** overexpression in **the fat body** | ♂ | Control | 100 | 83.5 | 87.0 | 24.0 | 102.0 | 81.0 | 91.0 | 70.0 | 95.0 | 197.1 | 14.0 | 1.4 |  |  |
|  |  | Mutant | 100 | 51.5 | 51.0 | 4.0 | 96.0 | 40.0 | 63.0 | 33.5 | 70.5 | 264.0 | 16.2 | 1.6 | **P < 0.0001** | **P < 0.001** |
|  |  | Control | 100 | 85.3 | 88.0 | 38.0 | 108.0 | 76.0 | 94.0 | 66.0 | 101.0 | 208.2 | 14.4 | 1.4 |  |  |
|  |  | Mutant | 100 | 53.3 | 51.0 | 2.0 | 96.0 | 41.0 | 71.0 | 25.0 | 83.5 | 443.6 | 21.1 | 2.1 | **P < 0.0001** | **P < 0.001** |
|  | ♀ | Control | 100 | 79.8 | 80.0 | 8.0 | 115.0 | 71.0 | 91.0 | 59.0 | 99.5 | 294.6 | 17.2 | 1.7 |  |  |
|  |  | Mutant | 100 | 69.3 | 75.0 | 4.0 | 107.0 | 58.5 | 87.0 | 29.5 | 91.0 | 595.4 | 24.4 | 2.4 | **P = 0.0023** | **P < 0.05** |
|  |  | Control | 100 | 75.6 | 79.0 | 3.0 | 105.0 | 66.5 | 89.0 | 52.5 | 96.5 | 352.5 | 18.8 | 1.9 |  |  |
|  |  | Mutant | 100 | 67.5 | 70.0 | 2.0 | 107.0 | 52.0 | 85.0 | 39.0 | 93.5 | 482.1 | 22.0 | 2.2 | **P = 0.0042** | **P < 0.01** |
| ***sgg-RB A81T*** overexpression in **the fat body** | ♂ | Control | 100 | 83.5 | 87.0 | 24.0 | 102.0 | 81.0 | 91.0 | 70.0 | 95.0 | 197.1 | 14.0 | 1.4 |  |  |
|  |  | Mutant | 100 | 57.3 | 54.0 | 10.0 | 103.0 | 49.0 | 64.5 | 44.0 | 78.5 | 221.4 | 14.9 | 1.5 | **P < 0.0001** | **P < 0.001** |
|  |  | Control | 100 | 85.3 | 88.0 | 38.0 | 108.0 | 76.0 | 94.0 | 66.0 | 101.0 | 208.2 | 14.4 | 1.4 |  |  |
|  |  | Mutant | 100 | 64.6 | 63.0 | 35.0 | 106.0 | 55.0 | 72.0 | 45.5 | 84.5 | 223.8 | 15.0 | 1.5 | **P < 0.0001** | **P < 0.001** |
|  | ♀ | Control | 100 | 79.8 | 80.0 | 8.0 | 115.0 | 71.0 | 91.0 | 59.0 | 99.5 | 294.6 | 17.2 | 1.7 |  |  |
|  |  | Mutant | 100 | 95.7 | 98.0 | 41.0 | 123.0 | 90.0 | 105.0 | 78.0 | 110.5 | 241.3 | 15.5 | 1.6 | **P < 0.0001** | **P < 0.001** |
|  |  | Control | 100 | 75.6 | 79.0 | 3.0 | 105.0 | 66.5 | 89.0 | 52.5 | 96.5 | 352.5 | 18.8 | 1.9 |  |  |
|  |  | Mutant | 100 | 91.2 | 95.5 | 46.0 | 116.0 | 85.5 | 103.0 | 63.5 | 107.0 | 259.5 | 16.1 | 1.6 | **P < 0.0001** | **P < 0.001** |
| ***sgg-RB Y214F*** overexpression in **the nervous system** | ♂ | Control | 100 | 69.1 | 75.0 | 8.0 | 92.0 | 58.5 | 81.5 | 44.5 | 90.0 | 325.5 | 18.0 | 1.8 |  |  |
|  |  | Mutant | 100 | 40.8 | 42.0 | 6.0 | 74.0 | 36.5 | 45.0 | 28.0 | 52.0 | 107.8 | 10.4 | 1.0 | **P < 0.0001** | **P < 0.001** |
|  |  | Control | 100 | 80.9 | 83.0 | 4.0 | 108.0 | 76.0 | 94.0 | 59.0 | 101.0 | 374.1 | 19.3 |  | 1,9 |  |
|  |  | Mutant | 100 | 53.3 | 54.0 | 14.0 | 90.0 | 47.0 | 62.0 | 25.0 | 76.0 | 290.8 | 17.1 | 1.7 | **P < 0.0001** | **P < 0.001** |
|  | ♀ | Control | 100 | 73.1 | 77.0 | 7.0 | 96.0 | 70.0 | 82.0 | 58.5 | 86.0 | 260.4 | 16.1 | 1.6 |  |  |
|  |  | Mutant | 100 | 70.9 | 71.0 | 39.0 | 84.0 | 65.0 | 77.0 | 61.5 | 82.5 | 71.6 | 8.5 | 0.8 | **P = 0.0002** | **P < 0.001** |
|  |  | Control | 100 | 88.8 | 94.0 | 8.0 | 111.0 | 81.0 | 100.0 | 67.5 | 101.0 | 244.9 | 15.6 | 1.6 |  |  |
|  |  | Mutant | 100 | 82.0 | 86.0 | 17.0 | 107.0 | 78.0 | 92.5 | 56.0 | 98.0 | 345.4 | 18.6 | 1.9 | **P = 0.0002** | **P < 0.001** |
| ***sgg-RB A81T*** overexpression in **the nervous system** | ♂ | Control | 100 | 69.1 | 75.0 | 8.0 | 92.0 | 58.5 | 81.5 | 44.5 | 90.0 | 325.5 | 18.0 | 1.8 |  |  |
|  |  | Mutant | 100 | 40.1 | 43.0 | 4.0 | 87.0 | 32.0 | 48.0 | 20.5 | 60.0 | 233.4 | 15.3 | 1.5 | **P < 0.0001** | **P < 0.001** |
|  |  | Control | 100 | 80.9 | 83.0 | 4.0 | 108.0 | 76.0 | 94.0 | 59.0 | 101.0 | 374.1 | 19.3 | 19.3 | 1,9 |  |
|  |  | Mutant | 100 | 53.5 | 55.0 | 9.0 | 78.0 | 48.0 | 64.0 | 30.5 | 70.5 | 250.0 | 15.8 | 1.6 | **P < 0.0001** | **P < 0.001** |
|  | ♀ | Control | 100 | 73.1 | 77.0 | 7.0 | 96.0 | 70.0 | 82.0 | 58.5 | 86.0 | 260.4 | 16.1 | 1.6 |  |  |
|  |  | Mutant | 100 | 61.3 | 67.0 | 12.0 | 83.0 | 54.5 | 76.0 | 29.0 | 79.5 | 363.6 | 19.1 | 1.9 | **P < 0.0001** | **P < 0.001** |
|  |  | Control | 100 | 88.8 | 94.0 | 8.0 | 111.0 | 81.0 | 100.0 | 67.5 | 101.0 | 244.9 | 15.6 | 1.6 | 1,9 |  |
|  |  | Mutant | 100 | 77.9 | 86.0 | 16.0 | 109.0 | 60.0 | 94.0 | 41.5 | 99.0 | 469.1 | 21.7 | 2.2 | **P < 0.0001** | **P < 0.01** |

Different pairs Control-Mutant of the same genotype and sex represent the results of independent experiments. Lifespans of control genotypes were published in [9]. Full description of genotypes is given in the Materials and Methods section. Significant (after Bonferroni corrections when appropriate) P-values are in bold case.
