# Supplementary material for: Modulated Expression of the Protein Kinase GSK3 in Motor and Dopaminergic Neurons Increases Female Lifespan in Drosophila melanogaster
Source: Front Genet. 2020 Jun 30;11:668. doi: 10.3389/fgene.2020.00668 (PMC7339944; doi:10.3389/fgene.2020.00668)
Supplement: Supplementary file 5 [file Table_2.DOCX]

Table S2. Parameters of maximum lifespan and Gompertz function in transgenic females with the increased lifespan.

| Effects | Sex | Genotype | Percentile 90. days | P values for comparisons with control genotype. Wang-Allison test | R_0_* | α* | MRDT** |
| --- | --- | --- | --- | --- | --- | --- | --- |
| ***sgg-RB A81T*** overexpression in **the fat body** | ♀ | Control | 99.5 |  | 0.0002040 | 0.0659473 | 10.47 |
|  |  | Mutant | 110.5 | **P = 0.0083** | 0.0000668 | 0.0677926 | 10.19 |
|  |  | Control | 96.5 |  | 0.0004563 | 0.0585489 | 11.79 |
|  |  | Mutant | 107.0 | **P = 0.0022** | 0.0000786 | 0.0687836 | 10.04 |
| ***sgg-RB Y214F*** overexpression in **motor** neurons | ♀ | Control | 83.0 |  | 0.0001997 | 0.0833725 | 8.28 |
|  |  | Mutant | 90.0 | **P = 0.0025** | 0.0001167 | 0.0798689 | 8.64 |
|  |  | Control | 82.0 |  | 0.0003464 | 0.0740483 | 9.32 |
|  |  | Mutant | 89.5 | **P = 0.0053** | 0.0001804 | 0.0758081 | 9.10 |
| ***sgg-RB A81T*** overexpression in **dopaminergic** neurons | ♀ | Control | 89.0 |  | 0.0001445 | 0.0765379 | 9.01 |
|  |  | Mutant | 106.5 | **P = 0.0362** | 0.0000899 | 0.0700355 | 9.85 |
|  |  | Control | 91.0 |  | 0.0001181 | 0.08860844 | 7.84 |
|  |  | Mutant | 107.0 | **P = 0.0022** | 0.0003262 | 0.06755638 | 10.22 |

Different pairs Control-Mutant of the same genotype and sex represent the results of independent experiments. Lifespans of control genotypes were published in [9]. Full description of genotypes is given in the Materials and Methods section. Significant P-values are in bold case.

* R_0,_ α - Gompertz equation parameters.

** MRDT - mortality rate doubling time.
