# Supplementary material for: Modulated Expression of the Protein Kinase GSK3 in Motor and Dopaminergic Neurons Increases Female Lifespan in Drosophila melanogaster
Source: Front Genet. 2020 Jun 30;11:668. doi: 10.3389/fgene.2020.00668 (PMC7339944; doi:10.3389/fgene.2020.00668)
Supplement: Supplementary file 6 [file Table_3.DOCX]

Table S3. Distributive statistics of the lifespan of transgenic flies with mutant *shaggy*-*RB* overexpression and *sgg* RNA-i knockdown in different neurons.

| Effects | Sex | Genotype | N | Mean | Median | Minimum | Maximum | Lower Quartile | Upper Quartile | Percentile 10 | Percentile 90 | Variance | Standard Deviation | Standard Error | P values for comparisons with control genotype | |
| --- | --- | --- | --- | --- | --- | --- | --- | --- | --- | --- | --- | --- | --- | --- | --- | --- |
|  |  |  |  |  |  |  |  |  |  |  |  |  |  |  | Mann-Whitney Test | Kolmogorov-Smirnov Test |
| ***sgg-RB Y214F*** overexpression in **peptidergic** neurons | ♂ | Control | 100 | 56.4 | 58.5 | 12.0 | 84.0 | 45.0 | 73.0 | 24.5 | 76.5 | 355.9 | 18.9 | 2.7 |  |  |
|  |  | Mutant | 100 | 59.8 | 61.5 | 13.0 | 80.0 | 54.0 | 68.0 | 46.0 | 74.0 | 179.0 | 13.4 | 1.9 | P = 0.3857 | P > 0.10 |
|  | ♀ | Control | 100 | 77.4 | 84.0 | 6.0 | 101.0 | 72.5 | 90.0 | 53.0 | 95.0 | 434.0 | 20.8 | 2.1 |  |  |
|  |  | Mutant | 100 | 77.7 | 80.0 | 27.0 | 102.0 | 72.0 | 87.0 | 58.5 | 91.0 | 188.7 | 13.7 | 1.4 | P = 0.1600 | P > 0.10 |
| ***sgg-RB A81T*** overexpression in **peptidergic** neurons | ♂ | Control | 100 | 56.4 | 58.5 | 12.0 | 84.0 | 45.0 | 73.0 | 24.5 | 76.5 | 355.9 | 18.9 | 2.7 |  |  |
|  |  | Mutant | 100 | 52.0 | 57.5 | 10.0 | 81.0 | 45.0 | 65.0 | 19.5 | 72.0 | 343.6 | 18.5 | 2.6 | P = 0.2263 | P > 0.10 |
|  | ♀ | Control | 100 | 77.4 | 84.0 | 6.0 | 101.0 | 72.5 | 90.0 | 53.0 | 95.0 | 434.0 | 20.8 | 2.1 |  |  |
|  |  | Mutant | 100 | 77.9 | 84.0 | 8.0 | 110.0 | 63.5 | 93.0 | 49.0 | 100.5 | 448.2 | 21.2 | 2.1 | P = 0.6797 | P > 0.10 |
| ***sgg-RB Y214F*** overexpression in **cholinergic** neurons | ♂ | Control | 100 | 70.2 | 78.5 | 7.0 | 104.0 | 57.0 | 86.0 | 38.0 | 92.0 | 475.7 | 21.8 | 3.1 |  |  |
|  |  | Mutant | 100 | 63.9 | 60.5 | 17.0 | 100.0 | 52.0 | 81.0 | 39.0 | 87.5 | 391.6 | 19.8 | 2.8 | P = 0.0431 | P < 0.05 |
|  | ♀ | Control | 100 | 73.7 | 81.0 | 7.0 | 109.0 | 52.0 | 98.0 | 33.0 | 103.5 | 734.9 | 27.1 | 3.8 |  |  |
|  |  | Mutant | 100 | 65.4 | 67.0 | 14.0 | 104.0 | 55.0 | 82.0 | 24.0 | 96.0 | 565.1 | 23.8 | 3.4 | P = 0.0579 | P < 0.05 |
| ***sgg-RB A81T*** overexpression in **cholinergic** neurons | ♂ | Control | 100 | 70.2 | 78.5 | 7.0 | 104.0 | 57.0 | 86.0 | 38.0 | 92.0 | 475.7 | 21.8 | 3.1 |  |  |
|  |  | Mutant | 100 | 71.9 | 80.0 | 10.0 | 102.0 | 63.0 | 84.0 | 34.5 | 88.5 | 415.3 | 20.4 | 2.9 | P = 0.9450 | P > 0.10 |
|  | ♀ | Control | 100 | 73.7 | 81.0 | 7.0 | 109.0 | 52.0 | 98.0 | 33.0 | 103.5 | 734.9 | 27.1 | 3.8 |  |  |
|  |  | Mutant | 100 | 76.0 | 80.0 | 12.0 | 108.0 | 70.0 | 92.0 | 40.5 | 102.5 | 560.0 | 23.7 | 3.3 | P = 0.9890 | P > 0.10 |
| ***sgg-RB Y214F*** overexpression in **glutamatergic** neurons | ♂ | Control | 100 | 78.8 | 81.0 | 8.0 | 107.0 | 70.0 | 87.0 | 62.0 | 95.0 | 243.0 | 15.6 | 1.6 |  |  |
|  |  | Mutant | 100 | 54.9 | 57.0 | 17.0 | 73.0 | 48.0 | 66.0 | 40.5 | 70.5 | 146.2 | 12.1 | 1.2 | **P < 0.0001** | **P < 0.001** |
|  | ♀ | Control | 100 | 73.4 | 76.0 | 3.0 | 94.0 | 67.0 | 83.5 | 58.5 | 89.0 | 261.9 | 16.2 | 1.6 |  |  |
|  |  | Mutant | 100 | 76.4 | 81.0 | 14.0 | 93.0 | 71.0 | 86.0 | 59.0 | 89.0 | 185.5 | 13.6 | 1.4 | P = 0.1228 | P < 0.10 |
| ***sgg-RB A81T*** overexpression in **glutamatergic** neurons | ♂ | Control | 100 | 78.8 | 81.0 | 8.0 | 107.0 | 70.0 | 87.0 | 62.0 | 95.0 | 243.0 | 15.6 | 1.6 |  |  |
|  |  | Mutant | 100 | 66.8 | 66.5 | 40.0 | 90.0 | 59.0 | 78.0 | 51.0 | 83.0 | 147.4 | 12.1 | 1.2 | **P < 0.0001** | **P < 0.001** |
|  | ♀ | Control | 100 | 73.4 | 76.0 | 3.0 | 94.0 | 67.0 | 83.5 | 58.5 | 89.0 | 261.9 | 16.2 | 1.6 |  |  |
|  |  | Mutant | 100 | 76.6 | 78.0 | 11.0 | 97.0 | 74.0 | 83.5 | 64.0 | 90.0 | 192.6 | 13.9 | 1.4 | P = 0.1187 | P < 0.10 |
| ***sgg-RB Y214F*** overexpression in **GABA-ergic** neurons | ♂ | Control | 100 | 77.1 | 81.0 | 30.0 | 95.0 | 72.0 | 87.0 | 59.0 | 88.0 | 149.0 | 12.2 | 1.2 |  |  |
|  |  | Mutant | 100 | 71.4 | 73.0 | 46.0 | 88.0 | 66.5 | 79.0 | 57.0 | 81.0 | 86.0 | 9.3 | 0.9 | **P < 0.0001** | **P < 0.001** |
|  | ♀ | Control | 100 | 67.0 | 66.0 | 26.0 | 93.0 | 59.0 | 78.0 | 50.0 | 83.0 | 184.7 | 13.6 | 1.4 |  |  |
|  |  | Mutant | 100 | 67.0 | 66.5 | 28.0 | 104.0 | 54.5 | 78.0 | 46.5 | 88.0 | 254.1 | 15.9 | 1.6 | P = 0.8069 | P > 0.10 |
| ***sgg-RB A81T*** overexpression in **GABA-ergic** neurons | ♂ | Control | 100 | 77.1 | 81.0 | 30.0 | 95.0 | 72.0 | 87.0 | 59.0 | 88.0 | 149.0 | 12.2 | 1.2 |  |  |
|  |  | Mutant | 100 | 69.6 | 72.0 | 30.0 | 93.0 | 65.0 | 76.5 | 58.0 | 81.0 | 120.1 | 11.0 | 1.1 | **P < 0.0001** | **P < 0.001** |
|  | ♀ | Control | 100 | 67.0 | 66.0 | 26.0 | 93.0 | 59.0 | 78.0 | 50.0 | 83.0 | 184.7 | 13.6 | 1.4 |  |  |
|  |  | Mutant | 100 | 68.7 | 67.0 | 25.0 | 98.0 | 59.5 | 81.0 | 50.0 | 88.0 | 223.5 | 14.9 | 1.5 | P = 0.3898 | P > 0.10 |
| ***sgg-RB Y214F*** overexpression in **motor** neurons | ♂ | Control | 100 | 68.0 | 72.0 | 16.0 | 92.0 | 59.0 | 78.0 | 47.5 | 81.5 | 178.4 | 13.4 | 1.3 |  |  |
|  |  | Mutant | 100 | 55.6 | 55.0 | 7.0 | 69.0 | 45.0 | 62.0 | 42.5 | 64.0 | 123.5 | 11.1 | 1.1 | **P < 0.0001** | **P < 0.001** |
|  |  | Control | 100 | 58.6 | 61.0 | 14.0 | 81.0 | 54.5 | 66.0 | 49.0 | 70.5 | 131.6 | 11.5 | 1.1 |  |  |
|  |  | Mutant | 100 | 47.6 | 47.0 | 25.0 | 65.0 | 42.0 | 53.0 | 38.0 | 59.0 | 67.8 | 8.2 | 0.8 | **P < 0.0001** | **P < 0.001** |
|  |  | Control | 50 | 64.0 | 70.5 | 8.0 | 89.0 | 56.0 | 77.0 | 34.5 | 84.0 | 392.0 | 19.8 | 2.8 |  |  |
|  |  | Mutant | 50 | 49.8 | 51.5 | 7.0 | 75.0 | 45.0 | 61.0 | 21.5 | 69.0 | 275.6 | 16.6 | 2.3 | **P < 0.0001** | **P < 0.001** |
|  | ♀ | Control | 100 | 69.0 | 70.5 | 20.0 | 92.0 | 60.5 | 80.0 | 52.0 | 83.0 | 200.1 | 14.1 | 1.4 |  |  |
|  |  | Mutant | 100 | 78.4 | 80.0 | 46.0 | 95.0 | 72.0 | 84.0 | 65.0 | 90.0 | 88.6 | 9.4 | 0.9 | **P < 0.0001** | **P < 0.001** |
|  |  | Control | 100 | 65.4 | 70.5 | 17.0 | 83.0 | 59.5 | 75.0 | 41.0 | 82.0 | 235.4 | 15.3 | 1.5 |  |  |
|  |  | Mutant | 100 | 76.3 | 78.0 | 19.0 | 96.0 | 68.0 | 86.0 | 61.5 | 89.5 | 160.6 | 12.7 | 1.3 | **P < 0.0001** | **P < 0.001** |
|  |  | Control | 50 | 68.0 | 68.5 | 41.0 | 88.0 | 59.0 | 80.0 | 51.0 | 83.0 | 150.4 | 12.3 | 1.7 |  |  |
|  |  | Mutant | 50 | 76.0 | 81.0 | 30.0 | 97.0 | 70.0 | 84.0 | 49.0 | 89.5 | 217.1 | 14.7 | 2.1 | **P = 0.0007** | **P < 0.001** |
| ***sgg-RB A81T*** overexpression in **motor** neurons | ♂ | Control | 100 | 68.0 | 72.0 | 16.0 | 92.0 | 59.0 | 78.0 | 47.5 | 81.5 | 178.4 | 13.4 | 1.3 |  |  |
|  |  | Mutant | 100 | 54.8 | 57.0 | 11.0 | 79.0 | 49.0 | 60.5 | 41.5 | 68.5 | 136.8 | 11.7 | 1.2 | **P < 0.0001** | **P < 0.001** |
|  |  | Control | 100 | 58.6 | 61.0 | 14.0 | 81.0 | 54.5 | 66.0 | 49.0 | 70.5 | 131.6 | 11.5 | 1.1 |  |  |
|  |  | Mutant | 100 | 47.6 | 47.0 | 25.0 | 65.0 | 42.0 | 53.0 | 38.0 | 59.0 | 67.8 | 8.2 | 0.8 | **P < 0.0001** | **P < 0.001** |
|  | ♀ | Control | 100 | 69.0 | 70.5 | 20.0 | 92.0 | 60.5 | 80.0 | 52.0 | 83.0 | 200.1 | 14.1 | 1.4 |  |  |
|  |  | Mutant | 100 | 69.2 | 74.0 | 4.0 | 100.0 | 59.5 | 83.0 | 42.0 | 91.5 | 387.8 | 19.7 | 2.0 | P = 0.4539 | P > 0.10 |
|  |  | Control | 100 | 65.4 | 70.5 | 17.0 | 83.0 | 59.5 | 75.0 | 41.0 | 82.0 | 235.4 | 15.3 | 1.5 |  |  |
|  |  | Mutant | 100 | 66.1 | 71.0 | 12.0 | 87.0 | 66.0 | 74.5 | 39.0 | 81.5 | 251.3 | 15.9 | 1.6 | P = 0.7573 | P > 0.10 |
| Strong *sgg* **knockdown** in **motor** neurons | ♂ | Control | Not analyzed | | | | | | | | | | | | | |
|  |  | Mutant | Lethal | | | | | | | | | | | | | |
|  | ♀ | Control | Not analyzed | | | | | | | | | | | | | |
|  |  | Mutant | Lethal | | | | | | | | | | | | | |
| Weak *sgg* **knockdown** in **motor** neurons | ♂ | Control | 100 | 76,6 | 79,0 | 9,0 | 107,0 | 69,0 | 86,0 | 54,0 | 94,0 | 285,8 | 16,9 | 1,7 |  |  |
|  |  | Mutant | 100 | 63,0 | 65,0 | 38,0 | 100,0 | 53,0 | 71,5 | 45,0 | 77,0 | 162,6 | 12,8 | 1,3 | **P < 0.0001** | **P < 0.001** |
|  | ♀ | Control | 100 | 79,5 | 82,0 | 26,0 | 101,0 | 75,0 | 90,0 | 61,0 | 93,0 | 197,1 | 14,0 | 1,4 |  |  |
|  |  | Mutant | 100 | 75,9 | 79,0 | 21,0 | 101,0 | 70,0 | 86,0 | 55,5 | 94,0 | 256,6 | 16,0 | 1,6 | P = 0,0892 | P > 0.10 |
| ***sgg-RB Y214F*** overexpression in **dopaminergic** neurons | ♂ | Control | 100 | 74.3 | 74.5 | 3.0 | 108.0 | 70.0 | 81.0 | 66.0 | 84.0 | 135.9 | 11.7 | 1.2 |  |  |
|  |  | Mutant | 100 | 67.8 | 68.0 | 20.0 | 90.0 | 65.0 | 76.0 | 53.5 | 82.5 | 157.4 | 12.5 | 1.3 | **P < 0.0001** | **P < 0.001** |
|  |  | Control | 100 | 66.8 | 70.0 | 27.0 | 99.0 | 60.0 | 72.0 | 43.5 | 86.0 | 197.5 | 14.1 | 1.4 |  |  |
|  |  | Mutant | 100 | 48.8 | 51.5 | 5.0 | 77.0 | 43.0 | 61.0 | 26.0 | 68.0 | 249.5 | 15.8 | 1.6 | **P < 0.0001** | **P < 0.001** |
|  | ♀ | Control | 100 | 79.7 | 82.0 | 40.0 | 99.0 | 72.0 | 86.0 | 67.5 | 89.0 | 90.6 | 9.5 | 1.0 |  |  |
|  |  | Mutant | 100 | 80.6 | 82.0 | 30.0 | 102.0 | 72.0 | 87.5 | 66.0 | 95.0 | 148.9 | 12.2 | 1.2 | P = 0.4606 | P > 0.10 |
|  |  | Control | 100 | 78.8 | 83.0 | 20.0 | 101.0 | 76.0 | 90.0 | 56.0 | 91.0 | 250.0 | 15.8 | 2.2 |  |  |
|  |  | Mutant | 100 | 76.3 | 81.5 | 19.0 | 101.0 | 69.0 | 89.0 | 61.5 | 90.5 | 297.2 | 17.2 | 2.4 | P = 0.3310 | P > 0.10 |
| ***sgg-RB A81T*** overexpression in **dopaminergic** neurons | ♂ | Control | 100 | 74.3 | 74.5 | 3.0 | 108.0 | 70.0 | 81.0 | 66.0 | 84.0 | 135.9 | 11.7 | 1.2 |  |  |
|  |  | Mutant | 100 | 66.8 | 66.0 | 45.0 | 95.0 | 60.0 | 70.0 | 58.0 | 82.0 | 84.0 | 9.2 | 0.9 | **P < 0.0001** | **P < 0.001** |
|  |  | Control | 100 | 66.8 | 70.0 | 27.0 | 99.0 | 60.0 | 72.0 | 43.5 | 86.0 | 197.5 | 14.1 | 1.4 |  |  |
|  |  | Mutant | 100 | 50.9 | 54.0 | 5.0 | 82.0 | 46.5 | 57.0 | 30.0 | 65.0 | 214.8 | 14.7 | 1.5 | **P < 0.0001** | **P < 0.001** |
|  |  | Control | 50 | 72.3 | 76.0 | 14.0 | 91.0 | 63.0 | 83.0 | 61.0 | 88.5 | 212.4 | 14.6 | 2.1 |  |  |
|  |  | Mutant | 50 | 56.8 | 61.0 | 23.0 | 83.0 | 49.0 | 67.0 | 37.0 | 69.0 | 190.9 | 13.8 | 2.0 | **P < 0.0001** | **P < 0.001** |
|  |  | Control* | 50 | 69.6 | 75.0 | 6.0 | 100.0 | 59.0 | 80.0 | 41.5 | 95.0 | 398.5 | 20.0 | 2.8 |  |  |
|  |  | Mutant* | 50 | 61.8 | 62.0 | 33.0 | 85.0 | 51.0 | 74.0 | 42.0 | 78.0 | 182.7 | 13.5 | 1.9 | **P = 0.0035** | **P < 0.025** |
|  | ♀ | Control | 100 | 79.7 | 82.0 | 40.0 | 99.0 | 72.0 | 86.0 | 67.5 | 89.0 | 90.6 | 9.5 | 1.0 |  |  |
|  |  | Mutant | 100 | 87.4 | 91.0 | 34.0 | 114.0 | 80.0 | 99.0 | 61.0 | 106.5 | 318.1 | 17.8 | 1.8 | **P < 0.0001** | **P < 0.001** |
|  |  | Control | 100 | 78.8 | 83.0 | 20.0 | 101.0 | 76.0 | 90.0 | 56.0 | 91.0 | 250.0 | 15.8 | 2.2 |  |  |
|  |  | Mutant | 100 | 91.2 | 95.0 | 41.0 | 109.0 | 83.0 | 102.0 | 70.0 | 107.0 | 221.6 | 14.9 | 2.1 | **P < 0.0001** | **P < 0.001** |
|  |  | Control | 50 | 71.4 | 74.0 | 37.0 | 97.0 | 64.5 | 79.0 | 56.0 | 85.5 | 134.3 | 11.6 | 1.2 |  |  |
|  |  | Mutant | 50 | 74.3 | 79.0 | 3.0 | 97.0 | 70.0 | 85.0 | 48.0 | 90.0 | 250.8 | 15.8 | 1.6 | **P = 0.0033** | **P < 0.001** |
|  |  | Control* | 50 | 62.8 | 63.5 | 13.0 | 98.0 | 49.0 | 82.0 | 31.5 | 88.0 | 437.8 | 20.9 | 3.0 |  |  |
|  |  | Mutant* | 50 | 82.1 | 86.0 | 9.0 | 108.0 | 76.0 | 94.0 | 63.5 | 100.5 | 384.2 | 19.6 | 2.8 | **P < 0.0001** | **P < 0.001** |
| Strong *sgg* **knockdown** in **dopaminergic** neurons | ♂ | Control | 50 | 50,6 | 47,5 | 12,0 | 103,0 | 28,0 | 70,0 | 23,50 | 85,0 | 608,9 | 24,6 | 3,4 |  |  |
|  |  | Mutant | 26 | 17,2 | 18,0 | 3,0 | 33,0 | 6,0 | 25,0 | 5,0 | 31,0 | 99,80 | 9,9 | 1,9 | **P < 0.0001** | **P < 0.001** |
|  | ♀ | Control | 50 | 76,2 | 82,0 | 24,0 | 110,0 | 53,0 | 97,0 | 33,5 | 108,0 | 714,4 | 26,7 | 3,7 |  |  |
|  |  | Mutant | 50 | 24,5 | 21,0 | 4,0 | 103,0 | 11,0 | 26,0 | 7,0 | 50,5 | 394,4 | 19,8 | 2,8 | **P < 0.0001** | **P < 0.001** |
| Weak *sgg* **knockdown** in **dopaminergic** neurons | ♂ | Control | 50 | 60,3 | 68,0 | 15,0 | 81,0 | 40,0 | 79,0 | 30,5 | 80,0 | 398,2 | 19,9 | 2,8 |  |  |
|  |  | Mutant | 50 | 54,0 | 60,0 | 18,0 | 67,0 | 45,0 | 65,0 | 31,5 | 67,0 | 200,9 | 14,1 | 2,0 | **P=0,0044** | **P < 0.001** |
|  | ♀ | Control | 49 | 91,5 | 89,0 | 82,0 | 106,0 | 86,0 | 98,0 | 83,0 | 101,0 | 51,7 | 7,1 | 1,0 |  |  |
|  |  | Mutant | 50 | 81,1 | 80,5 | 68,0 | 102,0 | 75,0 | 88,0 | 68,0 | 89,5 | 75,8 | 8,7 | 1,2 | **P < 0.0001** | **P < 0.001** |

Different pairs Control-Mutant of the same genotype and sex represent the results of independent experiments. Lifespans of control genotypes were published in [9]. Full description of genotypes is given in the Materials and Methods section. Significant (after Bonferroni corrections when appropriate) P-values are in bold case.

* The experiment with the *w*;* ***P{w[+mC]=UAS-mCD8::GFP.L}LL5/Cy;*** *P{w^+mC^=ple-GAL4.F}3* (D11) driver.
