# Supplementary material for: Modulated Expression of the Protein Kinase GSK3 in Motor and Dopaminergic Neurons Increases Female Lifespan in Drosophila melanogaster
Source: Front Genet. 2020 Jun 30;11:668. doi: 10.3389/fgene.2020.00668 (PMC7339944; doi:10.3389/fgene.2020.00668)
Supplement: Supplementary file 7 [file Table_4.DOCX]

Table S4. Distributive statistics of the lifespan of transgenic flies with aPKC knockdown in motor neurons.

| The line inducing RNA interference | Sex | Genotype | N | Mean | Median | Minimum | Maximum | Lower Quartile | Upper Quartile | Percentile 10 | Percentile 90 | Variance | Standard Deviation | Standard Error | P values for comparisons with control genotype | |
| --- | --- | --- | --- | --- | --- | --- | --- | --- | --- | --- | --- | --- | --- | --- | --- | --- |
|  |  |  |  |  |  |  |  |  |  |  |  |  |  |  | Mann-Whitney Test | Kolmogorov-Smirnov Test |
| **aPKC KD1** | ♂ | Control | 100 | 66.2 | 67.0 | 9.0 | 99.0 | 54.5 | 76.5 | 52.5 | 91.0 | 294.1 | 17.1 | 1.7 |  |  |
|  |  | Mutant | 100 | 53.3 | 55.0 | 13.0 | 84.0 | 45.5 | 61.0 | 37.5 | 68.5 | 162.7 | 12.8 | 1.3 | **P < 0.0001** | **P < 0.001** |
|  | ♀ | Control | 100 | 80.4 | 81.0 | 13.0 | 121.0 | 71.5 | 93.0 | 62.0 | 99.0 | 266.5 | 16.3 | 1.6 |  |  |
|  |  | Mutant | 100 | 76.8 | 80.0 | 28.0 | 121.0 | 69.0 | 86.0 | 57.5 | 92.0 | 232.2 | 15.2 | 1.5 | P = 0.0640 | P < 0.05 |
| **aPKC KD2** | ♂ | Control | 100 | 66.2 | 67.0 | 9.0 | 99.0 | 54.5 | 76.5 | 52.5 | 91.0 | 294.1 | 17.1 | 1.7 |  |  |
|  |  | Mutant | 100 | 50.4 | 50.5 | 19.0 | 77.0 | 46.0 | 59.0 | 28.0 | 65.0 | 169.9 | 13.0 | 1.3 | **P < 0.0001** | **P < 0.001** |
|  | ♀ | Control | 100 | 80.4 | 81.0 | 13.0 | 121.0 | 71.5 | 93.0 | 62.0 | 99.0 | 266.5 | 16.3 | 1.6 |  |  |
|  |  | Mutant | 100 | 77.0 | 80.0 | 10.0 | 101.0 | 70.5 | 87.0 | 59.0 | 94.0 | 225.1 | 15.0 | 1.5 | P = 0.0812 | P < 0.10 |

Full description of genotypes is given in the Materials and Methods section. Significant (after Bonferroni corrections when appropriate) P-values are in bold case.
